# Supplementary material for: Nabilone treatment for severe behavioral problems in adults with intellectual and developmental disabilities: Protocol for a phase I open-label clinical trial
Source: PLoS One. 2023 Apr 12;18(4):e0282114. doi: 10.1371/journal.pone.0282114 (PMC10096227; doi:10.1371/journal.pone.0282114)
Supplement: S1 File — (PDF) [file pone.0282114.s001.pdf]

Informed Consent Form for Participation in a Research Study

Study Title: **Phase I pre-pilot open-label clinical trial of nabilone for severe behavioural problems (aggression) in adults with intellectual and developmental disabilities (N-AND)**

Study Doctor: Hsiang-Yuan Lin, MD

Principal Investigator: Hsiang-Yuan Lin, MD
Staff Psychiatrist, Adult Neurodevelopmental and Geriatric Psychiatry Division
Clinician-Scientist, Azrieli Adult Neurodevelopmental Centre
Centre for Addiction and Mental Health
Assistant Professor of Department of Psychiatry
University of Toronto
416-535-8501 ext. 32817
Hsiang-Yuan.Lin@camh.ca; n-and@camh.ca

Sponsor: CAMH

Funders: The CAMH AFP Innovation Fund; Department of Psychiatry Excellence Funds

Contact Number: Dr. Hsiang-Yuan Lin by telephone at 416-535-8501 ext. 32817 (if there is nobody attending a call, please leave a voice message)

INTRODUCTION

As a Substitute Decision Maker, you are being to provide informed consent on behalf of a person (the 'participant') who cannot do so themselves. This means you'll receive education about a CAMH study, and you'll be able to use this information to decide if the participant will take part in the study. If the participant gains the ability to consent for themselves, then your role as a Substitute Decision Maker will end.

Throughout this form, the term "you" refers to the person you are representing. We assess your capacity to give informed consent at a screening visit before we begin the consent process and other study procedures, using a tool called the MacArthur Competence Assessment Tool for Clinical Research (MacCAT-CR).

In this document, you will find information about this study, including why it is being done, what you would be asked to do, benefits and potential risks, information about privacy and confidentiality, and who to contact with questions. This is called an informed consent form.

Because you expressed an interest in participating in this study, you are receiving a copy of this informed consent form in either paper or PDF format based on the distribution method you agreed to.

Once you have time to review the information in the consent form, we will meet with you to go over the content of this form together to ensure that you understand what you are signing up for. You will then be asked if you have any further questions. If you do not have questions, you will be asked if you would like to consent to participate in the study. This meeting of consent discussion will be conducted either in person or virtually (as explained on Page 6.

Please ask the research team any questions you have and to explain anything that you do not understand.

It is your choice whether to take part in this study or not. If you decide to participate, you can change your mind later. No matter what you decide, it will not affect the care or any other services that you receive at CAMH or any other institutes/clinics.

Please take as much time as you need to decide. If you'd like to, you can talk about this study with other people (for example, your family, friends, your usual doctor, and other health professionals).

IS THERE A CONFLICT OF INTEREST?

There are no conflicts of interest to declare related to this study. That is, the researchers on the team do not have certain relationships with any companies or organizations that would benefit them financially, or in any other way, from the outcome of this trial.

WHAT IS THIS STUDY?

This study is to test whether a medication called Nabilone can help some people feel calmer, sleep better, and be less aggressive, with potentially less negative side effects, like body weight gain, of other drugs.

One in five adults with developmental disabilities have a hard time managing their anxiety or frustration and can hurt themselves or other people when they are upset.

The first way to help with this is to understand what is happening for the person, and help to teach them

different ways to deal with being upset. This can be done with a doctor, psychologist, an OT (occupational therapist) or a behavioural therapist. Sometimes this is not enough to help the situation and a doctor might give a medication to try to stop the person from hurting other people or themselves.

A problem with these medications is that they can have a lot of side effects like gaining weight and causing tiredness, and they don't always work that well. Some people have to take new medications to help with the side effects of the first medications. It is important to find other ways to help people that are safe and that may not have as many side effects.

One possible solution is Nabilone, a medication that is similar to cannabis (marijuana/'weed'). It can help some people to feel calmer, feel less pain, and be less aggressive. Meanwhile, it does not make people feel 'high' like cannabis does.

Nabilone has helped the mood of older people suffering from dementia, people who have scary nightmares with post-traumatic stress disorder, and those with Parkinson's Disease who have problems with pain, anxiety, and sleep.

Health Canada makes the rules about what drugs can be bought by people at the drug store, and what drugs can be used in research to figure out if they will work for people for different reasons. Health Canada says Nabilone can be part of this research project to find out if it helps people with developmental disabilities who hurt themselves or other people.

This study is being done to see if it is safe for adults with developmental disabilities to take Nabilone. It is called a Phase 1 pre-pilot study. This means we are trialing the new medicine with a small number of people. It is the first time this drug will be used by people with developmental disabilities who hurt themselves or other people when they are upset. It is called a Phase 1 pre-pilot study.

We aim to find out how people feel being in a project like this, and what effect the nabilone has on them. If participants are comfortable being part of this project and continuing to take Nabilone, we'll advance the project to a Phase 2 pilot randomized control study, where we include more people. Just because you are part of this study doesn't necessarily mean you will be part of a later project with Nabilone, or that we will do a bigger project later.

WHAT OTHER CHOICES ARE THERE?

You do not have to take part in this study if you do not want to. You can keep getting help from your doctor (we call this 'usual care' because it is what you would usually do if you do not do the research project) even if you decide not to do it. You can also choose to take part in other research projects at CAMH.

Please talk to your usual doctor or the doctor in charge of this project (study doctor) to understand what would be helpful or hard about doing this project or doing something else instead.

HOW MANY PEOPLE WILL BE IN THIS PROJECT?

We are looking for 30 people to be in this CAMH study. This study should take 9 weeks from beginning to end.

WHAT WILL HAPPEN DURING THIS STUDY?

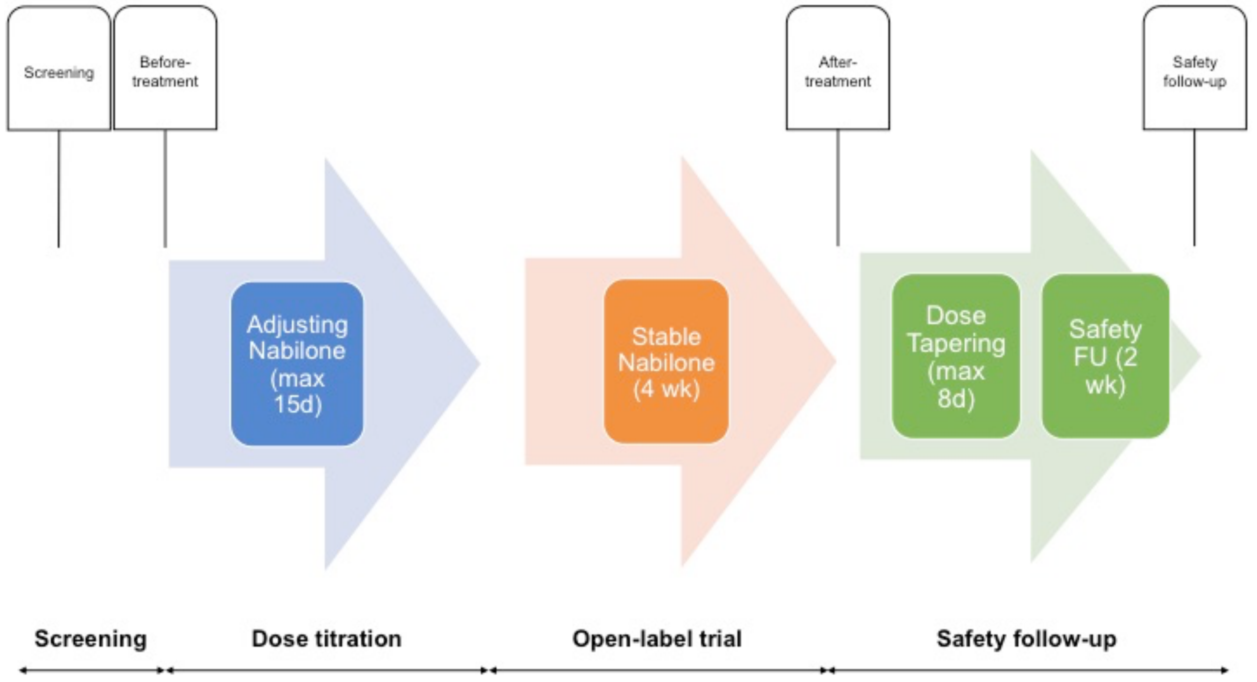

- Here is what will happen if you decide to be part of this project. There are three parts to it. You will meet with someone from someone on our team four times and these meetings will happen at the CAMH on Queen street. The first meeting will be about two hours, the second and third meetings will be about 3 hours, and the last meeting will be about two hours.

- Here is what will happen at Part 1 ("Screening" and "Before-treatment")
    - You will have two visits (#1 & 2) at CAMH to check if this study is a good fit for you, and to learn more about your health, how you feel, and your reactions to strong emotions like anxiety.
    - A psychiatrist will meet with you and talk to you about the medicines you take now and that you used to take, and about your current and past health. They will also take your blood pressure and see how fast your heart beats.
    - With your permission, we will ask your family doctor to send us information about blood tests you've done or if you had an EKG (a test that checks how your heart is functioning) in the last six months.
    - We will ask you some questions about your mood and how you've expressed your feelings in the last month.
    - You and your caregiver will complete some paper-and-pencil and computer tests to assess your mood, cognition (which means 'thinking'), and ability to function before the start of Nabilone use.
    - As part of the "before treatment" visit, you will come to CAMH to pick up the Nabilone for the adjusting Nabilone phase

- Here is what will happen when you are taking Nabilone ("Drug trial phase")

- We will first start by finding out what the best dose of Nabilone is for you (this may take up to 15 days). You will start with 0.25 mg of Nabilone once a day, and we will gradually increase your dose through phone calls with the study doctor every two days. Your dose will be adjusted until you notice that your mood and your response to anxiety and other strong emotions is getting better, or until we reach a maximum dose of 2 mg per day.
- After we figure out the best dose for you, you will take that amount of Nabilone for four weeks. You will come to CAMH again to return any unused or leftover Nabilone from the adjusting phase and pick up the Nabilone for the stable four weeks phase.

- Here is what will happen after the end of Nabilone use ("After-treatment" and "Safety follow-up").
    - You will have the other two visits (visits #3 and 4) to see how you are feeling after taking Nabilone and to see if you've had any side effects. Visit 3 is at the end of Nabilone use, and Visit 4 is two weeks after Visit 3.
    - You will come to CAMH at Visit 3 ("After Treatment") to return any unused or leftover Nabilone from the stable phase and pick up the Nabilone for the tapering phase.
    - We will then slowly and carefully help you to take a little bit less Nabilone every day until you are not taking it any more. We do this slowly stopping medication too fast can cause bad side effects (we call these 'withdrawal effects').
    - We will talk on the phone every other day while you are in the process of stopping the Nabilone. We will also have an in-person visit (called a safety follow-up visit) two weeks after you are finished taking the Nabilone medication.
    - You will come to CAMH at Visit 4 ("Safety Follow-Up Visit") to return any unused or leftover Nabilone from the tapering phase.

- You may be seen more often if the study doctor determines that this is necessary.

How Long is the Study?

- This study will take about nine weeks from the day you consent to join it. The part when you are taking the Nabilone medicine will take four weeks (one month).

Non-Experimental Procedures

Below, you'll find the activities we will do together to understand how you are thinking and feeling. These are called 'non-experimental procedures' because they are separate from the taking of the Nabilone, which is the experimental part. Everything you do in this study besides taking the Nabilone is called the non-experimental part.

Here are the things we will do:

- Check your body: Every time we see you, a doctor or a nurse will check your blood pressure and heart beat and do a check-up of how your body is doing.

- Check for side effects: We will check if you have had any side effects from the medication. This will be while you are taking the medication and after you have finished taking it.

- Check your thinking ('cognition'): We will check how you are thinking, remembering and understanding things by asking you to do some activities on an iPad (the app is called NIH Toolbox®

Cognition Battery). We will do this together at CAMH before you get the medication, while you are on the medication, and when you are finished taking it. This will tell us if the medication makes it harder or easier to think, remember, and understand things. It takes approximately one hour to complete this test.

- Check your response to distressful emotions: We will ask your parent or brother or sister to give us some information about how often you experience things like being upset, anxiety, or frustration, and how much that happens. This will be done before you start the medication and once you've begun taking it.

- Check what you think of the project: We will also ask you and your family how it felt to take part in this study.

- Check your brain: We can also look at what is happening in your brain by doing an MRI scan. If you agree to do an MRI scan or want to learn more about it, please check the other consent form for MRI scans.

The information you provide is for research purposes only. Some of the questions are personal. You can choose not to answer questions if you wish.

Even though you may have provided information on a questionnaire, these responses will not be reviewed by your health care team - if you wish them to know this information, please bring it to their attention.

<u>Participant Diaries</u>

You will be asked to keep a diary of when you take your study medication (Nabilone). We will ask you to write down how much you take each time and the exact time that you take it every day. When you are done taking the medication, we will ask you to give the diary to us.

<u>Option of Virtual Assessments</u>

- If you find that the traffic and time associated with your commute to CAMH is inconvenient, or if you consider it is unsafe to come on site during COVID, some assessments during the trial can be electively carried out virtually using videoconferencing software. These virtual assessments include interviews of your basic information, clinical history, diagnoses alongside medication uses, and adverse effects.

- Blank paper copies of the caregiver-rated questionnaires can also be electively sent to you via mail. You can send them back to us after you complete them. We will provide you the pre-paid postage envelop so you do not have to pay for the return mail.

- We will use the software Webex as our virtual meeting platform.
    - To use Webex, we need to send you an email explaining how to set up the software. The email will also include the instructions for how to login and join the online meeting. For this session, please try to find a quiet place where you will not be disturbed. Please use earphones if you can. It's a good idea to test out the system for a few minutes before the session to make sure that your internet connection, microphone, and speakers are all working.
    - To ensure online safety, do not share the email link with others or record the video session. During the interviews, it is recommended that you use your home computer or personal

device, and not a shared or work device. Additionally, please use a home (private) Wi-Fi network and not a free (public) Wi-Fi network for your internet connection. When you enter your display name in Webex, the name you enter will be visible to the researcher who will interview you.
    - It is important that you do not share the information that is discussed in the session with others. Please keep this session confidential. Again, do not share the email link with others or record the video session.
    - You or the research team can stop the session at any time, including if there are technical difficulties. If there are technical issues, one of our technical staff may join the call to provide support.
    - The virtual meeting sessions will not be recorded.

- Potential Online Risks:
  Like online shopping, teleconferencing/videoconferencing technology has some privacy and security risks. It is possible that information could be intercepted by unauthorized people (hacked) or otherwise shared by accident. This risk can't be completely eliminated. We want to make sure that you are aware of this.

Optional Research

The Researchers doing this study are interested in doing additional optional research in the future. You will be told about this and will be given an additional optional study consent form to read and sign if you want to take part in this. You may decide not to participate in the optional research and still participate in this main study.

WHAT ELSE DO I NEED TO KNOW ABOUT THE STUDY TREATMENT?

If you have side effects from the medication, the study doctor may change how much medication you get and make it less. If the side effects are intolerable even after you take less medication, then the study doctor will say you should not take it anymore, and you may be removed from the study.

While you are doing this project, we will not change other medications that you may be on or recommend any other types of activities to help you with your problems of getting angry, frustrated etc. This way, if you experience any changes, we'll know it was from the Nabilone and not from anything else.

WHAT DO I NEED TO DO IN THE STUDY?

If you choose to be part of this study, you will:

- Tell the study doctor about your current health problems.
- Tell the study doctor about all medications and supplements, including vitamins and herbals, and check with the study doctor before starting, stopping or changing any of these. This is for your safety as these may interact badly with the Nabilone the doctor gives you.
- Tell the study doctor if you are thinking about participating in another research study.
- Come to CAMH to pick up the study medication
- Return any unused study medication.
- Return any participant diaries or questionnaires that you take home to complete.

- Tell the study doctor if you become pregnant or father a child while participating in this study.
- Stop taking Nabilone after the open-label trial completes (washout period).
- Not share the Nabilone with anyone. The medication is for you alone, and must not be shared with others. If someone accidentally takes Nabilone, they should immediately notify the research team to monitor their safety.
- Complete the surveys about how you found being in this study even if you did not complete the study.

CAN I DECIDE TO STOP THE STUDY?

You can choose to stop being in this study (called withdrawing) at any time without having to say why. If you choose to stop, you should tell the study doctor or research staff.

You may be asked questions about how the study went and also have a short check up to make sure it is safe to stop taking the medication.

You may change your mind about us using information we collected from you at any time. If you do this, it also means you stop being in the study.

Information that is recorded before you withdraw will be used by the researchers for the purposes of the study, but no further information will be collected after you withdraw your permission.

CAN THE DOCTOR STOP THE STUDY EARLY?

The study doctor may stop you from being in the study early, and without your consent, for reasons such as:

- You can't handle taking Nabilone
- You can't do all the parts of the study, like the checking your body and your thinking, and the surveys
- New information shows that Nabilone is no longer good for you
- CAMH decides to stop the study
- The people who are in charge of this research (the Regulatory Authority/ies, for example, Health Canada or research ethics board) decide the study needs to stop
- If you plan to or become pregnant, or plan to give breastfeeding

If this happens, it may mean that you would not receive the medication for the full four weeks.

If you are removed from this study, the study doctor will explain why and make a plan for you to get help after the study.

WHAT ARE THE RISKS OR HARMS OF PARTICIPATING IN THIS STUDY?

You may experience side effects from participating in this study. Some side effects are known and are listed below, but there may be other side effects that are not expected. You should discuss these with the study doctor.

The study doctor will watch you closely to see if you have side effects. When possible, other medicine will be given to you to make side effects less serious and more tolerable. Many side effects go away shortly after the study treatment is stopped, but in some cases side effects can be serious, long-lasting, permanent, or may even cause death.

Based on previous research, the most commonly seen side effects of Nabilone and how likely they are to happen are listed here:

- Drowsiness (66.0%)
- Vertigo (58.8%)
- Psychological high (38.8%)
- Dry mouth (21.6%)
- Depression (14.0%)
- Ataxia (reduced body coordination) (12.8%)
- Blurred vision (12.8%)
- Sensation disturbance (12.4%)
- Anorexia (loss of appetite) (7.6%)
- Asthenia (feeling of numbness and tingling) (7.6%)
- Headache (7.2%)
- Orthostatic hypotension (feeling dizzy when standing up) (5.2%)
- Euphoria (4.0%)
- Hallucinations (2.0%).

The side effects of psychosis (2% for psychotic experiences. These experiences are not equal to full-blown psychotic disorders, of which the occurrence is theoretically expected low but the accurate rate is unknown) and abuse (getting addicted; <1%) are unlikely, and generally occur in the specific groups of people (those with history or family history of psychotic disorders and substance abuses, who are excluded from the screening phase).

These estimates are influenced by many factors such as drug dose, patient characteristics, detection technique, setting, and physician judgments, among others. Consequently, the side effects listed above are presented mainly to indicate how frequently adverse effects were reported in clinical studies conducted to evaluate the safety and efficacy of Nabilone across different situations.

To reduce the risk of dizziness and light-headedness, you should get up slowly when rising from a sitting or lying position.

Tell the study doctor right away if you have any serious side effects, including: fainting, unusual weakness, vision changes, or mental/mood changes.

It is possible that other drugs (prescription and non-prescription drugs), vitamins, or herbals can interact poorly with Nabilone. This can result in either the medication not working as expected or result in severe side effects.

Long term effects of the Nabilone used in this study are unknown at this time.

WHAT ARE THE REPRODUCTIVE RISKS?

The effects that Nabilone may have on an unborn baby (fetus) are unknown. You must not become pregnant or father a baby while taking Nabilone or for one month after the last dose. The study doctor will discuss family planning with you to ensure that you or your partner do not become pregnant or father a baby during the study. The study doctor will also ensure that from the start of last two normal menses before the trial to one month after the end of the trial, you or your partner are using effective methods of birth control, including abstinence or consistently and correctly taking the oral contraceptives.

Participants should not nurse (breastfeed) a baby while taking Nabilone and for 1 month after the last dose because the drugs used in this study might be present in breast milk and could be harmful to a baby.

WHAT ARE THE BENEFITS OF PARTICIPATING IN THIS STUDY?

If you agree to take part in this study, the experimental treatment may or may not be of direct benefit to you.

We hope the information learned from this study will help other people with intellectual and developmental disabilities (IDD) and what are sometimes called severe behavioural problems in the future.

PRIVACY AND CONFIDENTIALITY

If you agree to be a part of this study, we will collect some information about you and your health. We may ask you questions or look at your medical records. Information collected for this study may identify you when used alone (e.g. your name) or when combined with other available information about you.

The personal information or personal health information that may be collected, used, and stored in connection with this study could include your:

- Personal Information:
    - Name
    - Address
    - Phone number
    - Email address (to share the details of research appointment including the appointment date and directions, and to facilitate the option of virtual assessment)
    - Medical record number
    - OHIP (health card) number
    - Date of birth
    - Images (optional, i.e., MRI)
    - Information obtained from the cognitive test in our test's iPad APP
- Personal Health Information:
    - Dates and results of medical tests or procedures
    - Information about your psychological or educational reports
    - The results of the previous study-specific tests or procedures
    - Sex and/or gender
    - Race/ethnicity
    - The name of your family physician or other health care providers.

We will also make a note in your medical record at CAMH that you are taking part in this study. A

copy of this consent form will be added to your health record as well. These help your safety if you have a medical problem. This information could be shared with other doctors and hospitals in Ontario so that they have the information needed to treat you if necessary. If you are worried about this, or have any questions, please contact the Information and Privacy Office at 416-535-8501 ext. 33314 or by email at privacy@camh.ca.

We will keep information about you as private as possible.

When we write reports about this project, we will never use your name or any private details about you. We give you a research number, and everything we write about this project uses that number instead of your name. For instance, if you are Participant 3, we'll refer to you as Participant 3 in our writing instead of using your name.

We will keep a list with everyone's name and project number in a file drawer that we keep locked. We will put information about this project with your research number into the computer. Only people at CAMH can find that information, and there is a password to keep it extra private. Even though the likelihood that someone may identify you from the coded data is very small, the risk can never be completely eliminated.

There are a few situations that happen where information we get from you might get shared:

- Our hospital wants to make sure that we do an excellent job keeping everyone's information private and that we do a good job. The CAMH Research Quality Assurance team or CAMH Research Ethics Board may look at the information we have collected about you, including your medical record at CAMH. They will keep what they look at confidential.

- People from Health Canada keep an eye on the use of Nabilone and may come to CAMH to look at your study records (including personal information and personal health information) to check that the information collected for the study is correct and follows proper laws and guidelines.

- Sometimes, we share information from this study that does not have information about who each person is ('identifying information'), with other researchers, with your permission. This way, if we already got important information about you and they want the same information for the other study, you don't have to give it twice. Sometimes, we also share information for additional analyses called secondary analysis of data.

- It is your choice if your data will be shared with other research studies at or outside CAMH. Sometimes your information may be shared with researchers outside of Canada. The privacy laws outside of Canada are different and may not be as strict. To reduce the risk, study data that are shared outside of Canada will be coded (meaning they will not contain your name). There is a risk that someone could trace the information back to you. The chance that someone could do this is very small, but the risk may grow in future if people come up with new ways of tracing information back to people.

By signing this consent form, you agree to allow us to send your study data outside of Canada. Please initial below to let us know if you are okay with your data being shared.
____ Yes, I am agreeable to my data being shared
_____ No, I do not want my data being shared

We cannot guarantee that all information sent over e-mail is completely safe. You should not share private information about yourself using e-mail. You can let us know if you do not want to be contacted using e-mails. If you are worried about your health and it you need help quickly, please you shouldn't email us, you should call your doctor. If it is an emergency, you should call 911. By being in this study, you are agreeing to allow the research team to use e-mail/text messages to contact you for the study.

WILL FAMILY DOCTORS/HEALTH CARE PROVIDERS KNOW WHO IS PARTICIPATING IN THIS STUDY?

Your CAMH doctor/family doctor/health care provider will not be told by the research team that you are taking part in the study, but you can tell them if you'd like to.

WILL INFORMATION ABOUT THIS STUDY BE AVAILABLE ONLINE?

A description of this clinical trial will be available on http://www.clinicaltrials.gov. This website will not include information that can identify you. At most, the website will include a summary of the results. You can search this website at any time.

WHAT IS THE COST TO PARTICIPANTS?

Nabilone will be supplied at no charge while you take part in this study.

You may not be able to receive the Nabilone after your participation in the study is completed. There are several possible reasons for this, some of which are:

- The treatment may not turn out to be effective or safe.
- Your caregivers may not feel it is the best option for you.
- You may decide it is too expensive and insurance coverage may not be available.

If you benefit from Nabilone, the study doctor will inform your primary physician and discuss the possibility of continuation of Nabilone after the end of study. The study doctor will talk to you about your options. But we are not offering the compassionate use of nabilone after the end of the study. The primary doctor can still prescribe this medication in an off-label way, because of the efficacy shown in the trial.

Participation in this study will not involve any additional costs to you or your private health care insurance.

ARE STUDY PARTICIPANTS PAID TO BE IN THIS STUDY?

If you decide to participate in this study, you will receive $200 ($20/hour for the onsite visit) after the completion of the study.

If you decide to leave the study, you will receive a payment based on the time you participate in the study.

If you get injured or have side effects from participation in this study, medical care will be provided in the same way you would normally get medical care (for example, by going to your family doctor or seeking emergency medical treatment if needed). The costs of your medical treatment will be paid for by the provincial medical plan or by seeking reimbursement from your private medical insurer (if any) to the extent that such coverage is available. There may be extra costs that are not covered by provincial insurance or your private medical plan. Examples of these extra costs could be medications or other needed medical treatments to treat side effects that you may experience. If you have private health care insurance, the insurer may not pay for these added costs.

WHAT ARE THE RIGHTS OF PARTICIPANTS IN A RESEARCH STUDY?

You will be told, in a timely manner, about new information that may be relevant to your willingness to stay in this study.

You have the right to be informed of the results of this study once the entire study is complete. If you would like to be informed of the results of this study, please let the study doctor know.

Your rights to privacy are legally protected by federal and provincial laws that require safeguards to ensure that your privacy is respected.

By signing this form you do not give up any of your legal rights against the study doctor, sponsor or involved institutions for compensation, nor does this form relieve the study doctor, sponsor or their agents of their legal and professional responsibilities.

You will be given a copy of this signed and dated consent form prior to participating in this study.

WHAT IF RESEARCHERS DISCOVER SOMETHING ABOUT A RESEARCH PARTICIPANT?

During the study, the researchers may learn something about you that they didn't expect. For example, the researchers may find out that you have another medical or psychiatric condition.

If any new clinically important information about your health is obtained as a result of your participation in this study, you will be given the opportunity to decide if you want to know that information.

WHOM DO PARTICIPANTS CONTACT FOR QUESTIONS?

If you have questions about further information regarding the trial and the rights of trial subjects, or if you are in the event of study-related injury, you can contact Dr. Hsiang-Yuan Lin and study team by telephone at 416-535-8501 ext. 32817 or by email at n-and@camh.ca or Hsiang-Yuan.Lin@camh.ca. If there is nobody attending the call, please leave a voice message.

If you have questions about your rights as a participant or about ethical issues related to this study, you can talk to someone who is not involved in the study at all. That person is the Chair of the Research Ethics Board (REB). The REB is a group of people responsible for the ethical oversight of this study. The Chair of the REB can be reached by telephone at 416-535-8501 ext. 34020.

SIGNATURES

- All of my questions have been answered,
- I understand the information within this informed consent form,
- I allow access to medical records and related personal health information as explained in this consent form,
- I do not give up any legal rights by signing this consent form,
- I agree that the research team may contact me by email/text for the purposes of this study,
- I agree, or agree to allow the person I am responsible for, to take part in this study.

_______________________________ ____

Signature of Participant/ Substitute Decision-Maker | PRINTED NAME | Date

If consent is provided by Substitute Decision Maker: _______________________________

PRINTED NAME of Participant

_______________________________ ____

Signature of Person Conducting the Consent Discussion | PRINTED NAME & ROLE | Date

The following attestation must be provided if the participant is unable to read or requires an oral translation:

If the participant is assisted during the consent process, please check the relevant box and complete the signature space below:

☐ The person signing below acted as an interpreter, and attests that the study as set out in the consent form was accurately sight translated and/or interpreted, and that interpretation was provided on questions, responses and additional discussion arising from this process.

______________________________	____________________	__________________
PRINT NAME<br>of Interpreter	Signature	Date

____
Language

☐ The consent form was read to the participant. The person signing below attests that the study as set out in this form was accurately explained to the participant, and any questions have been answered.

______________________________	____________________	__________________
PRINT NAME<br>of witness	Signature	Date

____
Relationship to Participant

Please note: More information regarding the assistance provided during the consent process should be noted in the medical record for the participant if applicable, noting the role or relationship of the impartial witness.
