# Supplementary material for: Nabilone treatment for severe behavioral problems in adults with intellectual and developmental disabilities: Protocol for a phase I open-label clinical trial
Source: PLoS One. 2023 Apr 12;18(4):e0282114. doi: 10.1371/journal.pone.0282114 (PMC10096227; doi:10.1371/journal.pone.0282114)
Supplement: S2 File — (PDF) [file pone.0282114.s002.pdf]

Assent Form for Participation in a Research Study

Study Title: Phase I pre-pilot open-label clinical trial of nabilone for severe behavioural problems (aggression) in adults with intellectual and developmental disabilities (N-AND)

Study Doctor: Hsiang-Yuan Lin, MD

Principal Investigator: Hsiang-Yuan Lin, MD
Staff Psychiatrist, Adult Neurodevelopmental and Geriatric Psychiatry Division
Clinician-Scientist, Azrieli Adult Neurodevelopmental Centre
Centre for Addiction and Mental Health
Assistant Professor of Department of Psychiatry, University of Toronto
416-535-8501 ext. 32817
Hsiang-Yuan.Lin@camh.ca; n-and@camh.ca

CONTACT NUMBER: 416-535-8501 ext. 32817 (if there is nobody attending the call, please leave a voice message)

<u>What is this research study about?</u>

This research study is a way to learn more about intellectual and developmental disabilities and how to treat severe behavioral problems. You do not have to be in this research study if you don't want to.

<u>Why am I being asked to be in this study?</u>

We are trying to learn more about using a medication to treat severe behavioral problems in people who have intellectual and developmental disabilities. We are inviting you to be in the study because you have an intellectual or developmental disability, and also have issues with agitation or aggression.

<u>Who will know I am in this study?</u>

Only the research team and your partner/family member/caregiver will know that you are in the study. There may be times where your family doctor will ask for results from tests that happen during this study, but we will only send them your results after asking you if we can do this

<u>If I join this study what do I need to do?</u>

If you join the study, your participation may last up to 9 weeks. We will ask you and your partner/family member/caregiver to come in 4 times, including this visit now (screening visit), a visit before starting the medication trial, a visit at the end of the medication trial, and follow-up visit, to answer questions and talk to us about how you are feeling. We will talk about ways to support you and your partner/family member/caregiver. Throughout the study, we will also call you on the phone to check in on you and your partner/family member/caregiver.

If you participate in the study, you will be given the study pill to take, and we will check the side effects and if the dose is good for you for the first 2 weeks. If the study pill and dose is good for you, you will be

given a study pill to take every day for 4 weeks, up to 1 pill twice each day. In between each in-person visits, we will call you to check how you are doing. We will continue to check on you throughout the study, and at the end we will check whether you still have any problems 2 weeks after stopping the study pill.

There are two extra, optional visits to scan your brain before and after the medication trial. This part of the study is optional, and you can still take part in the rest of the study even if you choose not to do the brain scans.

Will any part of the study hurt, or are there risks?
You may get some side effects from taking part in this study, such as feeling sleepy, having a dry mouth, feeling dizzy, or feeling nauseous. There may be some other side effects that we don't know about yet. You should discuss any of these symptoms with us and with the study doctor. We check for side effects at each visit to help us make sure that the pill is safe for you to take throughout the study.

Will the study help me?
If you take part in this study, the treatment may or may not help you. Some benefits that may happen are: helping out with medical research, getting support from us for both you and your partner/family member/caregiver, and your agitation becoming less.

Will the study help others?
We hope that the information we learn from this study will help other people with agitation and aggression in intellectual and developmental disabilities in the future.

Can I say no?
Yes of course, you can decide not to be in the study. It's up to you. No one will be upset if you don't want to do this study. You can talk to your partner/family member/caregiver about this if you are not sure about participating. If you do join the study, you can change your mind later and stop being part of it at any time.

What choices do I have if I say no to this study?
You do not have to take part in this study in order to receive treatment or care. Right now, there is no standard medication used to treat agitation and aggression in people with intellectual and developmental disabilities. However, you can discuss other treatment options with your personal doctor or study doctor. There may be other research studies that you can participate in. You can ask us for more information about these other choices.

Who will see information about me?
The information collected about you during this study will be kept safe and your name will not be kept with this information. Instead, a code will be used on our records instead of your name. The people doing the research will be able to see the information collected about you.

We may send results to your family doctor, only after we ask you and you say it's okay for us to do this. If you tell us that you plan to harm yourself or someone else, we may need to tell someone.

Do I get anything for being in the study?

You and your partner/family member/caregiver will receive $200 ($20/hour for the onsite visits), or $280 (if you also do the extra brain scans) after the study is finished, for your time, and travel.

What if I have questions?

You can ask any questions you want about the study. If you have any question about this study after you leave today, you can talk to your partner/family member/caregiver and also call the study doctor at 416-535-8501 ext. 32817. If there is nobody attending the call, please leave a voice message.

If you want to talk to someone who is not part of the study about your rights as a research participant, you can call the Chairs of the CAMH Research Ethics Board at 416-535-8501 ext. 34020.

You will be given a copy of this paper to keep.

Assent: This research study has been explained to me and I agree to be in this study.

I agree to take part in the optional brain scans (MRI). ____ Yes ____ No

____ ____________________

Participant's signature for assent Date

____

Participant's printed name

☐ Assent was obtained orally

Signature of person obtaining assent: I confirm that I have explained the study to the extent compatible with the participant's understanding, and that the participant has agreed to be in the study.

____ ____________________

Signature of person obtaining assent Date

____

Printed name of person obtaining assent
