# Supplementary material for: Nabilone treatment for severe behavioral problems in adults with intellectual and developmental disabilities: Protocol for a phase I open-label clinical trial
Source: PLoS One. 2023 Apr 12;18(4):e0282114. doi: 10.1371/journal.pone.0282114 (PMC10096227; doi:10.1371/journal.pone.0282114)
Supplement: S3 File — (PDF) [file pone.0282114.s003.pdf]

Acceptability/Feedback Questionnaire

Participant Identifier: ____________________

Thank you for your participation, we would like to get some feedback from you on the study. Please take a few moments to answer the following questions by either ticking the boxes or writing out your responses.

1. How did you feel about the way we approached you for your loved one (your family member) to participate in the study?

☐ Very Unsatisfied ☐ Unsatisfied ☐ Neutral ☐ Satisfied ☐ Very Satisfied

2. What did you think about the number of study visits?

☐ Too few ☐ Just right ☐ Too many

3. How did you feel about the length of each study visit?

☐ Too short ☐ Just right ☐ Too long

If you have additional comments for questions 2 and 3, please note them down below

4. How did you find the length of all the questionnaires?

☐ Too short ☐ Just right ☐ Too long

For the following statements below, please tick whether you strongly disagree, disagree, are neutral, agree, or strongly agree.

5. The questions in the questionnaire were easy to understand.

☐ Strongly Disagree ☐ Disagree ☐ Neutral ☐ Agree ☐ Strongly Agree

6. I enjoyed participating in this study.

☐ Strongly Disagree ☐ Disagree ☐ Neutral ☐ Agree ☐ Strongly Agree

7. Why did you want to sign up for this study?

8. What did you find most helpful about the study?

9. What did you find most difficult about the study?

10. Did you feel supported throughout the study?

☐ No ☐ Somewhat ☐ Yes

11. How could we have better supported you throughout the study?

12. Are there any other ways that we can improve the study?

13. Would you recommend this study to other families whose loved ones have severe behavioural problems (aggression)?

☐ No ☐ Maybe ☐ Yes

14. Is there anything else that you would like to tell us?
