# Supplementary material for: Nabilone treatment for severe behavioral problems in adults with intellectual and developmental disabilities: Protocol for a phase I open-label clinical trial
Source: PLoS One. 2023 Apr 12;18(4):e0282114. doi: 10.1371/journal.pone.0282114 (PMC10096227; doi:10.1371/journal.pone.0282114)
Supplement: S4 File — (PDF) [file pone.0282114.s004.pdf]

SPIRIT

STANDARD PROTOCOL ITEMS: RECOMMENDATIONS FOR INTERVENTIONAL TRIALS

SPIRIT 2013 Checklist: Recommended items to address in a clinical trial protocol and related documents*

Section/item	Item No	Description
**Administrative information**		
Title	1	Phase I pre-pilot open-label clinical trial of nabilone for severe behavioural problems (aggression) in adults with intellectual and developmental disabilities (N-AND)
Trial registration	2a	**Clinicaltrials.gov identifier**: NCT05273320
	2b	(see table below)

Data category	Information
Primary registry and trial identifying number	**Clinicaltrials.gov identifier**: NCT05273320
Date of registration in primary registry	March 10, 2022
Secondary identifying numbers	**CAMH REB#:** 135/2020<br>**Health Canada Control#:** 256517
Source(s) of monetary or material support	This project is funded by the CAMH Innovation Fund of the Alternative Funding Plan for the Academic Health Sciences Centres of Ontario, and the University of Toronto Department of Psychiatry Excellence Funds. H-YL is supported by is supported by the Azrieli Adult Neurodevelopmental Centre at Centre for Addiction and Mental Health, and an Academic Scholar Award from the Department of Psychiatry, University of Toronto.
Primary sponsor	Centre for Addiction and Mental Health
Secondary sponsor(s)	N/A
Contact for public queries	Hsiang-Yuan Lin, MD (416) 535-8501<br>Study Principal/Qualified Investigator<br>hsiang-yuan.lin@camh.ca<br>Centre for Addiction and Mental Health
Contact for scientific queries	Hsiang-Yuan Lin, MD<br>Centre for Addiction and Mental Health

Public title	Phase I Open-label Clinical Trial of Nabilone for Aggression in Adults With Intellectual and Developmental Disabilities (N-AND)
Scientific title	Phase I Pre-pilot Open-label Clinical Trial of Nabilone for Severe Behavioural Problems (Aggression) in Adults With Intellectual and Developmental Disabilities
Countries of recruitment	Canada
Health condition(s) or problem(s) studied	Severe behavioural problems in adults with intellectual and developmental disabilities (IDD)
Intervention(s)	**Experimental: Open-label**<br>**Titration**: Nabilone p.o., increased in 0.25mg increments every 2 days to a maximum of 1mg b.i.d.<br>**Open-label:** Nabilone p.o. at maximum dose tolerated for 28 days<br>**Tapering**: Nabilone p.o. decreased in 0.25mg decrements per day<br>**Drug:** Nabilone capsules at a maximum dose of 1mg twice a day.
Key inclusion and exclusion criteria	**Inclusion criteria**<br>Participants of any sex or gender, race or ethnicity meeting all criteria listed below will be included in the study:<br>1) Aged ≥25 years, based on Health Canada recommendations to restrict medical cannabis use to this age (33).<br>2) Adults with a DSM-5 diagnosis of intellectual disabilities (ID) meeting: a. Full-scale IQ <75 on a standardized cognitive assessment reported in their prior medical record; b. A deficit in adaptive function in at least one activity of life, as estimated by the ABAS-3, as rated by the caregiver. For those whose verified records are not available, they are deemed eligible if they are connected with Disability Services Ontario, which is the access point for adult developmental disabilities services funded by the Ministry of Children, Community and Social Services in Ontario, Canada. People with ID and other developmental disabilities, e.g., autism, Down syndrome, genetic

	conditions such as Angelman syndrome, fragile X syndrome, Prader-Willi Syndrome, etc., will also be enrolled.<br>3) SBPs, including aggressive, disruptive, and/or self-injurious behaviors in any situation (home, day program, clinic, etc.), as defined by a score ≥18 on the Aberrant Behaviour Checklist-Irritability subscale (ABC-I), and a score ≥4 on the Clinical Global Impressions-Severity scale (61). A consistent pattern of frequent SBPs should occur for >3 months ≥1 time per week.<br>4) Sexually active women of child-bearing potential must have a negative urine pregnancy test at the screening visit.<br>5) Sexually active women of child-bearing potential must use an effective method of birth control at least from the start of the last two normal menses before the screening visit to one month after the end of the study (i.e., completion of the safety visit). The accepted methods of contraception include total sexual abstinence if it is the usual and preferred lifestyle, or consistently and correctly taking the oral contraceptive.<br>6) Blood test in the previous 12 months showing liver function test with the alanine transaminase (ALT) ≤3 times the upper limit of normal and bilirubin ≤2 times the upper limit of normal.<br>7) At least one month since participating in another investigational drug trial.<br><br>**Exclusion criteria**<br>1) History of hypersensitivity to any cannabinoid.<br>2)The presence of an unstable seizure disorder as defined by having not been seizure-free for at least 6 months or anticonvulsant treatment has not been stable for at least 4 weeks.<br>3) The presence of any clinically significant or unstable medical conditions, including cardiovascular, liver, kidney, pulmonary disease, or the presence of known congenital brain malformation, as per

	investigator assessment based on medical history and chart review.<br>4) The presence of a lifetime diagnosis of a psychotic disorder, bipolar disorder, or substance use disorder, or current diagnosis of major depressive disorder or dementia, based on past psychiatric history noted in the medical chart, as well as Moss-PAS (ID) at S-V.<br>5) Family history of a psychotic disorder.<br>6) Change in psychotropic medications less than 4 weeks prior to study drug (nabilone) use.<br>7) At the time of screening, each potential participant medication list will be checked for drugs that are known to have drug-drug interactions with nabilone. The following drugs and doses will serve as exclusions:<br>a. Currently on benzodiazepines at a dose more than the benzodiazepine equivalent to lorazepam 2 mg daily.<br>b. Currently on medical psychostimulant, including methylphenidate (>100 mg daily), lisdexamfetamine (>70 mg daily), amphetamine/dextroamphetamine (Adderall XR®, >50 mg daily), dextroamphetamine (Dexedrine®, >50 mg daily) at a dose exceeding their respective maximum doses (as shown in the bracket after each agent) to treat attention-deficit/hyperactivity disorder (ADHD) in adults, based on the Canadian ADHD Resource Alliance (CADDRA) guideline (73).<br>c. Currently on nonbenzodiazepine hypnotics, including zaleplon (>10 mg daily), zolpidem (>10 mg daily), and zopiclone (>7.5 mg daily), at a dose exceeding their respective suggested safety doses (as shown in the bracket after each agent), based on Canadian Recalls and Safety Alerts (https://healthycanadians.gc.ca/recall-alert-rappel-avis/).

	d. Currently on any opioid.<br>e. Currently on barbiturate.<br>f. Drinking any alcohol in the week prior to the screening visit.<br>g. Recreational use of any psychomimetic drugs in the week prior to the screening visit, including ketamine, lysergic acid diethylamide (LSD), 3,4-methylenedioxy-methamphetamine (MDMA), magic mushrooms, phencyclidine (PCP), salvia, gamma hydroxybutyrate (GHB), bath salts, and methamphetamine.<br>8) Currently taking other cannabinoids, such as CBD or medical cannabis, from another source, unless participants and/or their caregivers are willing to stop such use or this treatment for at least 4 weeks prior to entering the study.<br>9) Participants who might travel out of the area for a significant time during the study.<br>10) Recently are participating in another investigational drug trial.<br>11) Pregnancy.<br>12) Sexually active women of child-bearing potential intending to give breastfeeding or get pregnant.
Study type	Interventional<br>Primary purpose: Treatment<br>Study phase: Phase I<br>Interventional study model: Single group assignment<br>Number of arms: 1<br>Masking: none (open-label)<br>Allocation: N/A
Date of first enrolment	June 3, 2022
Target sample size	30
Recruitment status	Currently recruiting
Primary outcome(s)	1. Tolerability of nabilone<br>To evaluate tolerability, we hypothesize that 80% of participants will complete the study protocol.<br>2. Safety profile of nabilone<br>To evaluate safety, we hypothesize that nabilone will be satisfactorily

	tolerated and have a favourable safety profile in adults with IDD, as quantified by lower rates of adverse events (AEs)<br>than those reported in the literature of antipsychotics in IDD.<br>[Time frame: 7 weeks]
Key secondary outcomes	1. Changes in severe behavioural problems (SBP) while on nabilone and after.<br>We will explore changes in SBP pre- and post-treatment.<br>2. Brain changes on MRI after being on nabilone (optional)<br>We will optionally explore brain changes on MRI in participants pre- and post-treatment. (For participants who are willing and able to tolerate MRI scans).<br>[Time frame: 7 weeks]

Protocol version 3

Issue Date: Dec 20, 2021
Protocol Amendment Number: v3.0
Author(s): Hsiang-Yuan Lin, Elia Abi-Jaoude, Pushpal Desarkar, Wei Wang, Stephanie Ameis, Meng-Chuan Lai, Yona Lunsky, Tarek K. Rajji

Revision Chronology:

Dec 20, 2021	(a) Specified the procedures for how participants/caregivers would be receiving and returning the study medication; (b) specified the various time points where we will check and record concomitant medications.
Oct 5, 2021	(a) Added the cutoff of MacCAT-CAR and the rationale behind it; (b) specified the rationale of the up- and down-titration schedules; (c) added inclusion/exclusion criteria surrounding pregnancy and contraception; (d) specified a list of prohibited medications/substances or their maximum allowed doses in the inclusion/exclusion criteria.
July 10, 2021	Detailed the elective elements of the remote consent and remote assessments.
May 10, 2021	(a) Modified the project title; (b) updated the information about the funders and sponsor; (c) clarified the procedure of "research screening appointment"; (d) expanded the details of inclusion/exclusion criteria; (e)

	clarified the procedures of using NIH Toolbox; (f) specified which assessment can be flexibly implemented virtually; (g) specified the 'drug diary' information; (h) specified the details of the implementation of the MacCAT-CR; (i) added a figure for a schematic reading of the study procedure.

Funding	4	This project is funded by the CAMH Innovation Fund of the Alternative Funding Plan for the Academic Health Sciences Centres of Ontario, and the University of Toronto Department of Psychiatry Excellence Funds. H-YL is supported by is supported by the Azrieli Adult Neurodevelopmental Centre at Centre for Addiction and Mental Health, and an Academic Scholar Award from the Department of Psychiatry, University of Toronto.<br><br>Teva-nabilone (0.25 mg capsule and 1 mg capsule used in the study) is manufactured by Teva Canada Limited.
Roles and responsibilities	5a	Hsiang-Yuan Lin, Elia Abi-Jaoude, Pushpal Desarkar, Wei Wang, Stephanie Ameis, Meng-Chuan Lai, Yona Lunsky, Tarek K. Rajji<br><br>H-YL is the Principal/Qualified Investigator of the study, while EA-J, PD, WW, SA, M-CL, YL, TKR are co-investigators.
	5b	**Sponsor:** Centre for Addiction and Mental Health<br>**Sponsor's Reference:**<br>**Address:** 1025 Queen Street West<br>**Phone:** 416-535-8501 (calling from Greater Toronto Area)<br>1-800-463-2338 (toll-free)
	5c	This project is funded by the CAMH Innovation Fund of the Alternative Funding Plan for the Academic Health Sciences Centres of Ontario, and the University of Toronto Department of Psychiatry Excellence Funds. H-YL is supported by is supported by the Azrieli Adult Neurodevelopmental Centre at Centre for Addiction and Mental Health, and an Academic Scholar Award from the Department of Psychiatry, University of Toronto. The funders had no role in the design of the study; in the collection, analyses, or interpretation of data; in the writing of the manuscript, or in the decision to publish the protocol.
	5d	N/A
**Introduction**		
Background and rationale	6a	**Intellectual and developmental disabilities and aggression**<br>Approximately 1-3% of adults across the world have some form of intellectual and developmental disabilities (IDD) (1, 2), and 20-40% of them experience severe behavioral problems (SBPs), including self-injurious behavior, disruptive behavior, or aggression (3, 4), in the absence of a

psychiatric diagnosis. These SBPs are associated with emotion dysregulation, pain, and sleep problems (5). SBPs are major contributors to morbidity, functional impairments, missed opportunities for learning, reduced quality of life, and well-being challenges on families, health, education and disability sectors (6).

SBPs in adults with IDD are extremely difficult to cope with. Although environmental adjustments and psychosocial interventions are considered first-line treatments based on national and international guidelines (7, 8), these options are not effective for some people with IDD and SBPs. Antipsychotics and other psychotropic medications are thus prescribed for up to 70% of adults with IDD (9, 10), off-label, despite equivocal efficacy (11). People with IDD, relative to those without IDD, are at a higher risk of side effects of psychotropic agents and are less able to report side effects (12, 13). These side effects include weight gain, metabolic disturbances, neurological symptoms, and avoidable death (14). Furthermore, because of suboptimal responses to currently available medications, polypharmacy is commonly used for SBPs in adults with IDD (10), potentially leading to more adverse events (11). Thus, new and safer interventions are pressingly needed for this population.

The Endocannabinoid System

Neurophysiologically, the endocannabinoid system may be involved in SBPs and aggression through a complex interplay with other systems (15). Specifically, stimulated by Δ9-tetrahydrocannabinol (THC), the primary psychoactive ingredient in cannabis, the cannabinoid receptor 1 (CB1) modulates the activity of serotonergic, noradrenergic, dopaminergic, γ-aminobutyric acid (GABA)ergic, and glutamatergic neurotransmitters. CB1 is a potent modulator of myriads of cognitive and behavioral processes including learning, attention, emotion regulation, sleep structure, and pain perception (16). The cannabinoid receptor 2 (CB2), also activated by THC, is mainly localized in peripheral immune cells and is expressed in the brain during neuroinflammation. It has been postulated that neuroinflammatory processes alter the metabolism and functions of neurotransmitters, neuroendocrine activity and neural plasticity, which could lead to the development of neuropsychiatric symptoms (17). Neuroinflammatory markers have close connections with peripheral inflammatory cytokines (18), whose levels correlate with aggression in children with Prader-Willi Syndrome (60% of these children have IDD and those without IDD have some form of learning disabilities) (19), healthy adults (20), and people with intermittent explosive disorder (21). Further, CB1 (22, 23) or CB2 (24) knockout mice show increased aggression compared to wild-type mice. In this context, CB2 agonist appears to provide a neuroprotective effect on neuroinflammation (25). CB1 (26) and CB2 (24) agonists at lower doses have been shown to reduce aggression in preclinical studies. A theoretical review also suggests that CB1 modulation may be a future candidate for the treatment of self-injury (27).

Human functional MRI (fMRI) data suggest that at low doses, THC attenuates anxiety responses and reduces amygdala reactivity and its functional coupling with frontal regions during processing of stimuli with negative emotional content. Conversely, at higher doses, the effect of acute administration of THC becomes anxiogenic (28). In parallel, human proton magnetic resonance spectroscopy (1H MRS) studies indicate that the GABA and glutamate-glutamine balance, which is modulated by CB1, is associated with emotion regulation and related psychopathology (29). Taken together, this evidence suggests that agents which can activate CB1 and CB2 may help decrease SBPs and aggression in adults with IDD given the essential roles of endocannabinoid and neuroimmune systems in a multitude of behavioral functions associated with aggression.

Choice of comparators	6b	**Why Nabilone?**<br>Anecdotal reports (30) and preliminary research (31) suggest that medical cannabinoids may be useful to treat SBPs and aggression in individuals with IDD. However, despite high hopes held by families (32), the scientific evidence is still in a very early stage. Most studies to date focused on cannabidiol (CBD), as THC use has been associated with cognitive decrements, psychotic symptoms, and addiction in recreational marijuana users (33). However, the mechanism of action and appropriate dose range of CBD, which, unlike THC, has only a very low affinity for CB1 and CB2, remains elusive. Moreover, prior evidence suggests limited beneficial properties of CBD for aggression and risk factors associated with SBPs (34). Another option is the plant form of medical cannabis. However, individuals have variable sensitivity to different cannabis strains with varied combinations of THC, CBD, and other ingredients (35), making it difficult to investigate the effects of plant-form cannabis in a controlled trial setting. Therefore, identifying a cannabis derivative with a clearer mechanism and applicability for SBPs in IDD is needed.<br>Nabilone is a synthetic oral THC analogue that acts as a partial agonist on both CB1 and CB2 in humans. Thus, it mimics the effect of THC on aggression, emotion regulation, sleep, and pain, but with more predictable side effects, better safety profiles, and less euphoria (36). Clinical studies suggest that nabilone may be useful in alleviating agitation in patients with dementia (37, 38), nightmares in patients with post-traumatic stress disorder (PTSD) (39), non-motor symptoms (i.e., mood dysregulation, sleep, pain) in patients with Parkinson's disease (40), and core symptoms in those with anxiety and pain disorders (39). Preliminary studies suggest that another synthetic THC, dronabinol (a full agonist at CB 1 and CB2), may be effective in alleviating SBPs in youth with IDD (41, 42). However, dronabinol has been withdrawn from the Canadian market for unknown reasons. Moreover, nabilone, relative to dronabinol, has better bioavailability (43) and may be more effective in alleviating SBPs (37). Notably, nabilone may have fewer adverse mental health effects such as agitation, irritability and psychosis, because, like THC, it has relatively weak partial agonist activity at

		CB1 while most other synthetic cannabinoids (including dronabinol) exhibit full CB1 agonist properties (15, 44, 45).<br>Nabilone is indicated for severe nausea and vomiting associated with cancer chemotherapy and is commercially available in Canada. Its use is usually safe and satisfactorily tolerated. The most frequently reported adverse reactions in previous clinical trials are drowsiness, vertigo, mild psychological high, and dry mouth, depending on the dose and population (36-38, 40, 46, 47). The safety profile and rate of adverse events for nabilone are comparable to those reported in studies of CBD (48) and clonidine (49), and appear more favorable than what has been reported for antipsychotics (11, 13, 14). Importantly, the addictive potential of nabilone is very low (50) because it is associated with less euphoria, slower onset of action, and more difficult titration compared to smoking cannabis. In fact, nabilone has been shown to help with sustained abstinence from marijuana (51). The incidence of nabilone-induced psychotic symptoms is rare and has only been reported in people with a personal or family history of psychosis.
Objectives	7	**Study Objective:** Current evidence suggests that nabilone could be a promising treatment for SBPs in adults with IDD. However, the efficacy and safety profiles of nabilone in this population have yet to be studied. To enhance the current state of evidence for nabilone use to treat SBPs and aggression in adults with IDD, we propose to conduct the first phase I open-label clinical trial with the primary objective being to collect preliminary data on the tolerability and safety profile of nabilone in adults with IDD. To evaluate tolerability, we hypothesize that 80% of participants will complete the study protocol. To evaluate safety, we hypothesize that nabilone will be satisfactorily tolerated and have a favorable safety profile in adults with IDD, as quantified by lower rates of adverse events than those reported in the literature of antipsychotics treatment for SBPs in individuals with IDD. The explorative objective is to describe changes in SBPs and aggression in participants pre- to post-treatment.
Trial design	8	This study is a Phase I pre-pilot open-label trial of nabilone in adults with IDD and SBPs. As shown in Figure 1, the trial first includes a screening visit (S-V) for eligibility, then a baseline visit (V 0), followed by a dose-titration phase, and then a 4-week open-label phase at a stable dose. At the end of the open-label phase, participants will undergo a termination visit (V 1), after which nabilone will be tapered off over 8 days. Subsequently, 2 weeks after the full discontinuation of nabilone, participants will undergo the last follow-up visit (V-F) to ensure safety.<br><br>**Figure 1.** A schematic diagram of the trial design, including all study visits and timeline. Abbreviation: S-V: screening visit; V 0: baseline visit; V 1: visit at the end of open-label phase; V-F: follow-up visit to ensure safety; d: days; wk: weeks; FU: follow-up; max: maximum.

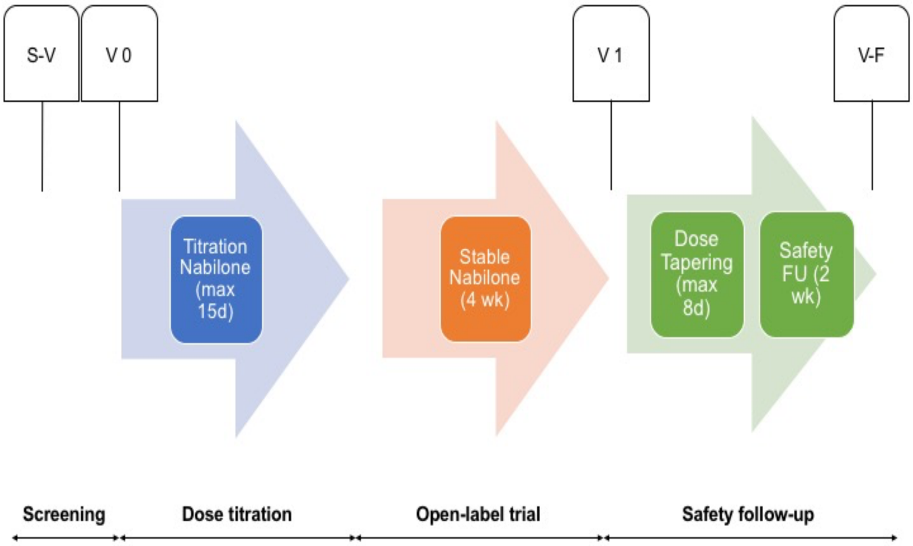

Methods: Participants, interventions, and outcomes

Study setting	9	All study visits will take place on-site at the Centre for Addiction and Mental Health. For the Screening Visit (S-V), all assessments will be done on-site. For future study visits (Pre-treatment, post-treatment, safety follow-up), interviews that collect data on demographics, clinical history, diagnoses alongside medication use, competence, and adverse effects can be conducted virtually using teleconferencing/videoconferencing software (Webex). Additionally, a blank paper copy of outcome measures rated by caregivers (including the ABC-I, MOAS, Anxiety-ADAMS, Stress-DASS-21, BFDS, and SCQ-AIQ) can be electively sent to them via mail. Completed questionnaires can be mailed, faxed, scanned, or photographed back to the study team. If the caregivers choose to send the completed questionnaire by mail, we will provide a pre-paid postage envelope.<br>The virtual sessions will be undertaken in coordination with in-person sessions based on the same research standards. The participants and caregivers can flexibly choose either the in-person or virtual assessments for those elective options (Table 1). We will notify both the participant and their caregiver of this virtual option when explaining the study (based on the Telephone Contact Script) and discussing the informed consent.
Eligibility criteria	10	**Inclusion criteria**<br>Participants of any sex or gender, race or ethnicity meeting all criteria listed below will be included in the study:<br>1) Aged ≥25 years, based on Health Canada recommendations to restrict medical cannabis use to this age (33).<br>2) Adults with a DSM-5 diagnosis of intellectual disabilities (ID) meeting: a. Full-scale IQ <75 on a standardized cognitive assessment reported in their prior medical record; b. A deficit in adaptive function in at least one activity of life, as estimated by the ABAS-3, as rated by the caregiver. For those

whose verified records are not available, they are deemed eligible if they are connected with Disability Services Ontario, which is the access point for adult developmental disabilities services funded by the Ministry of Children, Community and Social Services in Ontario, Canada. People with ID and other developmental disabilities, e.g., autism, Down syndrome, genetic conditions such as Angelman syndrome, fragile X syndrome, Prader-Willi Syndrome, etc., will also be enrolled.

3) SBPs, including aggressive, disruptive, and/or self-injurious behaviors in any situation (home, day program, clinic, etc.), as defined by a score ≥18 on the Aberrant Behaviour Checklist-Irritability subscale (ABC-I), and a score ≥4 on the Clinical Global Impressions-Severity scale (61). A consistent pattern of frequent SBPs should occur for >3 months ≥1 time per week.

4) Sexually active women of child-bearing potential must have a negative urine pregnancy test at the screening visit.

5) Sexually active women of child-bearing potential must use an effective method of birth control at least from the start of the last two normal menses before the screening visit to one month after the end of the study (i.e., completion of the safety visit). The accepted methods of contraception include total sexual abstinence if it is the usual and preferred lifestyle, or consistently and correctly taking the oral contraceptive.

6) Blood test in the previous 12 months showing liver function test with the alanine transaminase (ALT) ≤3 times the upper limit of normal and bilirubin ≤2 times the upper limit of normal.

7) At least one month since participating in another investigational drug trial.

Exclusion criteria

1) History of hypersensitivity to any cannabinoid.

2)The presence of an unstable seizure disorder as defined by having not been seizure-free for at least 6 months or anticonvulsant treatment has not been stable for at least 4 weeks.

3) The presence of any clinically significant or unstable medical conditions, including cardiovascular, liver, kidney, pulmonary disease, or the presence of known congenital brain malformation, as per investigator assessment based on medical history and chart review.

4) The presence of a lifetime diagnosis of a psychotic disorder, bipolar disorder, or substance use disorder, or current diagnosis of major depressive disorder or dementia, based on past psychiatric history noted in the medical chart, as well as Moss-PAS (ID) at S-V.

5) Family history of a psychotic disorder.

6) Change in psychotropic medications less than 4 weeks prior to study drug (nabilone) use.

7) At the time of screening, each potential participant medication list will be checked for drugs that are known to have drug-drug interactions with nabilone. The following drugs and doses will serve as exclusions:

a. Currently on benzodiazepines at a dose more than the benzodiazepine equivalent to lorazepam 2 mg daily.

		b. Currently on medical psychostimulant, including methylphenidate (>100 mg daily), lisdexamfetamine (>70 mg daily), amphetamine/dextroamphetamine (Adderall XR®, >50 mg daily), dextroamphetamine (Dexedrine®, >50 mg daily) at a dose exceeding their respective maximum doses (as shown in the bracket after each agent) to treat attention-deficit/hyperactivity disorder (ADHD) in adults, based on the Canadian ADHD Resource Alliance (CADDRA) guideline (73).<br>c. Currently on nonbenzodiazepine hypnotics, including zaleplon (>10 mg daily), zolpidem (>10 mg daily), and zopiclone (>7.5 mg daily), at a dose exceeding their respective suggested safety doses (as shown in the bracket after each agent), based on Canadian Recalls and Safety Alerts (https://healthycanadians.gc.ca/recall-alert-rappel-avis/).<br>d. Currently on any opioid.<br>e. Currently on barbiturate.<br>f. Drinking any alcohol in the week prior to the screening visit.<br>g. Recreational use of any psychomimetic drugs in the week prior to the screening visit, including ketamine, lysergic acid diethylamide (LSD), 3,4-methylenedioxy-methamphetamine (MDMA), magic mushrooms, phencyclidine (PCP), salvia, gamma hydroxybutyrate (GHB), bath salts, and methamphetamine.<br>8) Currently taking other cannabinoids, such as CBD or medical cannabis, from another source, unless participants and/or their caregivers are willing to stop such use or this treatment for at least 4 weeks prior to entering the study.<br>9) Participants who might travel out of the area for a significant time during the study.<br>10) Recently are participating in another investigational drug trial.<br>11) Pregnancy.<br>12) Sexually active women of child-bearing potential intending to give breastfeeding or get pregnant.
Interventions	11a	**Titration**: Nabilone p.o., increased in 0.25mg increments every 2 days to a maximum of 1mg b.i.d.<br>**Open-label:** Nabilone p.o. at maximum dose tolerated for 28 days<br>**Tapering**: Nabilone p.o. decreased in 0.25mg decrements per day
	11b	Reasons for withdrawing individual participants from the study may include one or more of the following:<br><br>a) Failure to continue to meet the inclusion criteria.<br>b) Changes in participants' behaviours or situations, which meet the exclusion criteria. For example, participants become pregnant, newly use other cannabinoids, or newly use illegal psychomimetic substances as specified in Section 3.3, after the start of the trial.<br>c) Major protocol violation.<br>d) Participant lost to follow-up.

e) Withdrawal of consent: Participation in the study is voluntary, and the participant can refuse or stop participation at any time. The participant's refusal to participate or withdraw from the study will not interfere in any way with the relationship with his/her doctor or hospital.
f) Inability to tolerate the study medication or procedure.
g) Serious adverse effects related to the study medication.
h) New information shows that the study intervention is no longer in the participant’s best interest.

In addition to these discontinuation criteria, any participant may be discontinued from the study at the discretion of the investigators if this is deemed to be in the best interest of the participant. The decision may be made either to protect the participant’s health and safety, or because it is part of the research plan that people who develop certain conditions may not continue to participate. To ensure safety, we will arrange a Safety Follow-up visit, which is the same as the one for those who fully complete the trials, for participants withdrawn from the study.

11c Caregivers will use the drug diary to record each administration of nabilone, including time, dosage, concomitant medications, and any noteworthy comments.

11d If participants fail to discontinue the use of any cannabinoids or high doses of benzodiazepine (more than the benzodiazepine equivalent to lorazepam 2 mg daily), psychostimulants for attention-deficit hyperactivity disorder (ADHD), and nonbenzodiazepine hypnotics 4 weeks before the start of the titration phase, they will not progress to the Pre-Treatment visit (V 0). We will discuss with participants and their caregivers to confirm their willingness to further proceed with the study. The participants are only permitted to proceed with the trial when they do not fulfill this exclusion criterion of the concurrent use of other cannabinoids and high dose of benzodiazepine.

If participants cannot, or are unwilling to, comply with the procedures of NIH Toolbox® Cognition Battery, but can comply with the remaining parts of the study, we will stop assessing them using this cognitive battery. These participants will still proceed with the other parts of the entire studies.
If participants have Adverse Events from participation in this study, medical care will be provided in the same way they would normally get medical care (for example, by going to their family doctor or seeking emergency medical treatment if needed)

Outcomes 12 **Outcome Measures**
The primary outcome measures are the adherence (as quantified by counting pill usage), adverse events based on the UKU side effect rating scale, and Crystallized and Fluid Composite scores of the NIH Toolbox® Cognition Battery. The exploratory outcomes encompass a range of clinical and neurophysiological measures (Table 1). Clinical measures include the

CGI, ABC-I, MOAS, and General Anxiety Subscale of ADAMS to quantify participants' behavioral changes, alongside changes in caregiver stress levels estimated with the Stress Subscale of the DASS-21 and the BFDS. All are reported by the caregivers, except the CGI.

MRI scans are optional and exploratory, dependent on participants' capacity to follow the procedures and the participants' and caregivers' willingness to participate. If a given participant and his/her/their caregiver and SDM are willing and capable to undergo an MRI scan, they will receive instructions using a custom-built social script at the screening visit (S-V). The social script comprises a series of photographs of the research procedures, environments, as well as audio files of the sounds emitted by the MRI scanner to help participants be acquainted with the scanning environment and procedures. We will use the CAMH research-dedicated 3 Tesla MRI scanner (GE MR750; General Electric, Milwaukee, WI). The acquisitions we will obtain include T1-weighted, a naturalistic film viewing functional MRI with negative emotion valence themes, and 1H MRS measuring neurometabolites including glutamate, glutamine, and myo-inositol at the anterior cingulate cortex (which is rich for CB1 and is involved in emotion regulation) scans. T1-weighted image and functional MRI will use Human Connectome Project-like data acquisition with multiband excitations (71) to reduce scan time, facilitating the successful scanning (72). The total estimated scanning time is 35 minutes.

Participant timeline 13

Screening visit (S-V)

Potential participants and their substitute decision-makers (SDMs) will attend a research screening visit. Before the informed consent process, the participant will be assessed for capacity to provide informed consent (see Supplementary File 1 for the Informed Consent Form). If he/she/they cannot pass the competence test using the MacArthur Competence Assessment Tool for Clinical Research (MacCAT-CR) (52), which provides a structured format for capacity assessment, then the written informed consent will be obtained from their SDM after seeking the participant's assent (see Supplementary File 2 for the Assent Form). A score of 70% or higher on the MacCAT-CR will indicate that a given participant is deemed competent to consent, given no relevant standard for people with IDD in the prior literature. This cut-off is in between the cut-offs used for adults with schizophrenia (53) and neurotypical children (54), which we deem to be a middle ground to balance sensitivity and specificity in assessing capacity to consent. In addition to the MacCAT-CR, the research team will also utilize clinical assessments of the potential participants to determine their capacity to consent to participate in this research, in a similar manner to determining capacity for clinical treatments as outlined in clinical guidelines (55). Participants will then be assessed for eligibility based on the inclusion and exclusion criteria (described below). We will also assess participants' other psychiatric conditions using the Moss Psychiatric Assessment Schedules (The Moss-PAS (ID), previously called Mini PAS-ADD) (56), and baseline adaptive

function using the caregiver-rated Adaptive Behavior Assessment System-III (ABAS-3), Adult Form (57).

Baseline visit (V 0)

At V 0 the following assessments will be completed:

- Clinical Global Impression – Severity scale (CGI-S) (58) will be rated by the investigator to evaluate the severity of SBPs.
- Baseline vital signs and weight.
- Participants' cognitive capacity using the NIH Toolbox® Cognition Battery (NIHTB-CB). Previous evidence suggests that nabilone may cause acute mild decrement in attention and working memory (37). We will use the NIHTB-CB, which has been shown as a good candidate measure to examine broad nonverbal cognitive changes for individuals with IDD (valid and reliable for those with a mental age of ≥5 years; implementable for those with a mental age of ≥3 years) (59), to assess the baseline attention and working memory.
- SBPs, including behavioral problems using the Aberrant Behavioral Checklist-Irritability subscale (ABC-I) (60, 61) and Modified Overt Aggression Scale (MOAS) (62), as reported by the caregivers. These scales will be the principal measures for the detecting changes in SBPs.
- Anxiety, using the parent-rated General Anxiety Subscale of the Anxiety, Depression, and Mood Scale (ADAMS) (63). Nabilone and THC at low doses have been shown to alleviate anxiety (39). Hence, this measure will be used for exploratory aims to detect a signal of changes in anxiety with nabilone use.
- Caregiver stress and crisis using the Stress Subscale of Depression Anxiety Stress Scales (DASS-21) (64) and Brief Family Distress Scale (BFDS) (65). As SBPs often poses major well-being challenges for caregivers, we aim to assess whether caregiver stress and distress improve if nabilone also improves SBPs of the participant.
- Autistic characteristics using the Social Communication Questionnaire for Adults with Intellectual Disability (SCQ-AID) (66). Prior evidence suggests that autistic characteristics may be associated with SBPs (67). We aim to explore whether baseline autistic characteristics modulate the treatment effects of nabilone.
- MRI Imaging (optional, dependent on the participant's capability to complete the scans, to be determined based on the opinions of the caregivers/parents and investigator).
- Concomitant medication(s).

Titration and open-label phases of nabilone treatment

After V 0, eligible participants will receive open-label nabilone starting with a dosage of 0.25 mg at bedtime. During the dose titration phase (which could last up to 15 days), nabilone will be titrated in 0.25 mg increments every two days, using a twice daily dosing schedule, after consultation with the study team during regular phone calls. This proposed up-titration schedule was decided based on a clinical trial of nabilone treatment for non-motor

symptoms in adults with Parkinson's disease (40). These regular phone calls will be carried out every two days during the whole dose titration phase until the participant reaches a stable dose for the open-label phase. The study team will also check whether the participant shows any physical or mental changes believed to be attributed to adverse events based on the UKU side effect rating scale (68), and advise the participant and his/her/their caregivers accordingly on the next step of titration. Dose adjustments are performed until the participant reaches the maximum permitted dose of 1 mg twice daily or experiences intolerable adverse events believed to be related to nabilone. If intolerable, the participant will use nabilone at the previous lower dose, entering the open-label phase. Note that the suggested maximum nabilone daily dose per the product monograph is 6 mg. However, considering the potential vulnerability of individuals with IDD, we chose to administer a maximum total of 2 mg/day in the current proposal, identical to the maximum dose used in clinical trials in patients with dementia (37) and Parkinson's disease (40). Moreover, nabilone is an analog of THC, which appears to reduce anxiety at low doses but becomes anxiogenic at high doses (69).

Participants will then be on a stable, optimized nabilone dosage for the four-week open-label phase, after which the trial ends with an on-site termination visit (V 1). During the open-label trial phase, the study team will call the participant and their caregivers once weekly to perform a basic check-up and to follow up with any questions or concerns related to the trial. If there are any changes suspected to be adverse events, the UKU side effect rating scale will be conducted during the telephone check-up. Caregivers will use the drug diary to record each administration of nabilone, including time, dosage, concomitant medications, and any noteworthy comments.

Termination (post-treatment) visit (V 1)

At V 1, the following post-treatment assessments will be completed:

- Adverse events using the UKU side effect rating scale, conducted by the investigator, to systemically investigate physical adverse events of nabilone. The UKU side effect rating scale is a short and easy-to-use instrument that captures the core dimensions of side effects in patients using psychotropic medications and is delivered by interviewing and observing the patient and their caregivers. In this study, we will adopt the UKU side effect rating scale specifically adjusted to adults with IDD, which consists of 35 out of the original 48 items (68).
- Vital signs (including blood pressure to check orthostatic hypotension) and weight to monitor the physical adverse events of nabilone.
- Participants' cognitive capacity using the NIH Toolbox® Cognition Battery, to monitor any acute cognitive change (considered as an adverse event) due to nabilone.
- Caregiver-rated ABC-I, MOAS, and General Anxiety Subscale of ADAMS. CGI-S and CGI-I will be rated by the investigator to evaluate the severity and improvement, respectively, of the SBPs after nabilone treatment.

These re-assessments are implemented for the exploratory study aim of assessing behavioral changes pre- to post-treatment of nabilone.

- Caregiver's stress and crisis using the Stress Subscale of DASS-21 and BFDS, to investigate changes in caregiver's stress and distress after the participant's nabilone treatment.
- Optional MRI Imaging for those who complete the pre-treatment MRI and are willing and able to undergo a second of MRI scan. This is to explore changes in brain function and structure pre- to post-treatment of nabilone.

Tapering phase and safety follow-up visit (S-F)

Nabilone will be tapered in 0.25 mg daily decrements to prevent acute withdrawal effects. This down-titration schedule was decided based on a conservative modification from a clinical trial using nabilone to treat behavioural and psychological symptoms of dementia (37). Phone calls will be held every other day during the dose-tapering phase to check whether there are any changes believed to be attributed to withdrawal effects. At the completion of the tapering of nabilone, there will be a final reconciliation of the nabilone dispensed, consumed and remaining, based on the drug diary and the remaining drug doses returned. This reconciliation will be logged on an accountability form and used to measure compliance.

A safety follow-up visit (S-F) will be scheduled after 2 weeks of full discontinuation of the study drug. During the S-F, vital signs, the UKU side effect rating scale and NIH Toolbox® will be assessed again to check for any residual adverse event. During the S-F, participants (if capable) and caregivers will complete a custom-designed feedback questionnaire (Supplementary File 3) regardingthe acceptability of all study components, including recruitment, withdrawing from the study, study visit attendance, protocol adherence and the time burden of completing questionnaires. This caregiver and participant perspective will guide the planning for a subsequent clinical trial for this population (70). This practice will help us understand barriers and how best to facilitate participation in medication trials for participants with IDD and their families.

Sample size	14	To test Hypotheses 1 (i.e., tolerability and adherence) and 2 (i.e., safety profiles), we aim to enroll 30 patients to enter the dose titration phase, consistent with common sample sizes for Phase I trials (74).<br>We assume the attrition rate of 25% from the dose titration phase to entering the open-label phase. To test behavioral changes at the group level (Exploratory Objective), assuming the effect size of Cohen's d 0.6, which is similar to that detected in the study investigating the effects of nabilone on agitation associated with dementia (37), this sample size of remaining participants (N=23) will achieve power = 0.79 with two-sided α-level set at 0.05 (G*Power 3.1).
Recruitment	15	The study team will inform the clinicians at the Adult Neurodevelopmental Services at CAMH and Surrey Place Centre about the study through an

email introduction, followed by online meetings. Adult Neurodevelopmental Services, CAMH provide first-time intake psychiatric assessments to about 300 adults with IDD annually. 30% of them (around 90 people annually) may be potentially eligible patients. The treating physician/clinical care team will not obtain consent. They may identify potential research participants and obtain verbal permission from these potential participants and their SDM for a member of the research team to approach them. Potential participants and their substitute decision maker (SDM) who indicate further interest in hearing more about the study and provide assent to be contacted by a member of the research team will engage in an informed consent process (as described in the Telephone Screening Script).

CLEARR will be used to recruit participants for this study. CLEARR stands for Clinical Engagement And Research Recruitment. This has been approved by CAMH's Medical Advisory Committee, Information and Privacy Office and acknowledged by REB. All new referrals will be reviewed by the CLEARR coordinator and CLEARR physician for eligibility to participate using minimal inclusion/exclusion criteria outlined. Once a patient is identified, the attending physician will be notified via outlook calendar invite or email that their patient may be eligible for the study. The attending physician will decide whether research is appropriate for the patient and if so, they will ask the patient for consent to be contacted regarding the research study. If the patient provides verbal consent to be contacted to receive more information about the study, the physician will connect the patient with the CLEARR coordinator or study research team who will further explain the study. No PHI will be given to the study research team prior to the patient's consent.

Recruitment in the community (self-referral) will be conducted online via CAMH Research Registry, social media channels (Facebook, Twitter, Linkedin, Instagram) and community organizations providing services for individuals with IDD and their families. Advertisements may also be posted on relevant Facebook and Linkedin, or Instagram pages or be retweeted through their Twitter accounts. The message outlines the fundamentals of the study. Potential participants/SDM will then attend a research screening visit to determine eligibility if they meet all of the following criteria.

All potential participants and their SDM will be given the option to participate. Participation in the study is voluntary. The decision to participate will not affect patients' receipt of treatment or clinical services. Participants and their SDM will be informed that they have the option of terminating their participation at any time, without consequence and that no new data will be collected on them. Any existing data will be anonymized.

Methods: Assignment of interventions (for controlled trials)

Allocation:		
Sequence generation	16a	N/A
Allocation concealment mechanism	16b	N/A
Implementation	16c	N/A
Blinding (masking)	17a	N/A
	17b	N/A
**Methods: Data collection, management, and analysis**		
Data collection methods	18a	**Assessment for Diagnosis**<br>The Moss Psychiatric Assessment Schedules (The Moss-PAS (ID)): The Moss-PAS (ID) (previously called Mini PAS-ADD) (56) provides a wide-spectrum mental health assessment primarily designed for people with intellectual disabilities who have limited language or reduced cognitive development. This semi-structured diagnostic interview, delivered by the PI, is fully compliant with DSM-5 and ICD-11. It consists of 86 items on a 4-point scale: 0 (symptom not present)—3 (symptom is severe). The interview is divided into seven subscales: Depression, Anxiety, Obsessive Compulsive Disorder, Mania, Psychosis, Unspecified disorder, and Autism. Approximately 30 min to complete.<br><br>**Scales to measure autistic symptoms**<br>Social Communication Questionnaire for Adults with Intellectual Disability (SCQ-AID) (56): The SCQ is a caregiver-rated dimensional measure of ASD symptom severity. The SCQ-AID is an adaptive version to improve the utility of the SCQ for adults with IDD. This newly developed algorithm has 24 items compared with the 40 items in the original instrument. <15 minutes to complete.<br><br>**Scales to measure cognitive function**<br>NIH Toolbox® Cognition Battery (NIHTB-CB): The NIHTB-CB is an iPad-based battery of brief memory, executive function, processing speed, and language tests, which was developed within the NIH Blueprint for Neuroscience Research. The NIHTB-CB has the potential to provide a highly standardized, objective, and scalable tool for use across laboratories and clinical trial sites and has been shown as a good candidate outcome measure to examine broad nonverbal cognitive changes for individuals with IDD (valid and reliable for those with a mental age of ≥5 years; implementable for those with a mental age of ≥3 years) (59). The NIHTB-CB will be used to monitor the change in cognitive performance pre-and post-treatment of nabilone. To allow for accurate scoring based on the established norm, the administration of NIHTB-CB will collect a set of

limited personal identification information, including sex, age, and education level, plus study ID. This assessment will be delivered on-site at pre-treatment (V 0), post-treatment (V 1), and safety follow-up (V-F). It takes approximately 60 minutes to complete each full assessment. It should be noted that this administration time is approximate considering the different levels of motivation and cooperativeness in participants. The norming version for neurotypical people aged 3-85 years is intended to be administered with a 30-minute duration (48).

Scale to measure adaptive function

Adaptive Behavior Assessment System-III (ABAS-3), Adult Form. The ABAS-3 is a measure of adaptive behaviour that has been validated in a number of psychiatric populations and has been used to assess functioning in neurodevelopmental disabilities (57). It provides an overall composite score of adaptive functioning, including subscores indexing social skills, conceptual and practical skills. Approximately 20 minutes to complete.

Scale to measure behavioural problems

1. Aberrant Behavioral Checklist-Irritability subscale (ABC-I): The ABC is a 58-item rating scale completed by a caregiver. It consists of 5 subscales. Among them, the Irritability subscale consists of 15 items. The ABC was empirically derived to assess treatment effects, and it has sound psychometric characteristics (60). Caregivers were asked to consider the participant's behaviour over the last four weeks at Screen/Baseline. Here we will only employ the ABC-I to confirm eligibility (presence of tantrums, aggression, self-injury) and to assess treatment effects (61). <10 minutes to complete.
2. Modified Overt Aggression Scale (MOAS): The MOAS (62) is a reliable measure used to measure behavioural problems in adults with IDD. It consisted of 4 items and is administered by the researcher's interview with the caregiver. It was used as the primary outcome in one of the largest RCT on aggression in this population (52). <5 minutes to complete.

Scale to measure anxiety

General Anxiety Subscale of the Anxiety, Depression, and Mood Scale (ADAMS): The ADAMS (63) is an informant-report instrument specifically developed for adults with IDD, consisting of five subscales. Herein, we will only use the General anxiety subscale, which consists of seven items that cover five of the diagnostic criteria or symptoms of anxiety. The score on the General anxiety subscale ranges from 0 to 21. <5 minutes to complete.

Scales to measure overall clinical impression

Clinical Global Impressions (CGI) scale: The CGI is a well-established research rating tool to provide a brief, stand-alone assessment of the clinician's view of the patient's global functioning prior to and after

initiating a study medication (54). The CGI-Severity (CGI-S) asks the clinician one question: “Considering your total clinical experience with this particular population, how mentally ill is the patient at this time?” which is rated on the following seven-point scale: 1=normal, not at all ill; 2=borderline mentally ill; 3=mildly ill; 4=moderately ill; 5=markedly ill; 6=severely ill; 7=among the most extremely ill patients. This rating is based upon the average severity of observed and reported symptoms, behaviour, and function in the past seven days. The CGI-Improvement (CGI-I) is for the clinician to compare the patient's overall clinical condition to the baseline visit and is rated on a seven-point scale: “Compared to the patient's condition at admission to the project [prior to medication initiation], this patient's condition is: 1=very much improved since the initiation of treatment; 2=much improved; 3=minimally improved; 4=no change from baseline (the initiation of treatment); 5=minimally worse; 6= much worse; 7=very much worse since the initiation of treatment.” <5 minutes to complete.

Scales to assess capacity to make informed consent
MacArthur Competence Assessment Tool for Clinical Research (MacCAT-CR): The MacCAT-CR (52) provides a structured format for capacity assessment. Beginning with project-specific disclosures to potential participants, the MacCAT-CR measures the four generally accepted components of decision-making competence: understanding, appreciation, reasoning, and the ability to express a choice. The informed consent of the participants who fail to pass this assessment will be completed by their substitute decision maker. Herein, a score of 70% or higher in the MacCAT-CR will indicate that a given participant is deemed competent to consent. This cutoff is borrowed from the protocol of an ongoing Brain Canada-funded study on elderly with dementia - “Standardizing Care for Neuropsychiatric Symptoms and Quality of Life in Dementia”. This cutoff of 70% is in-between the cut-offs used for schizophrenic adults (53) and neurotypical children (54), which we deem a middle ground to balance sensitivity and specificity of assessing capacity to consent. This should take approximately 15 minutes to complete.

In addition to using the MacCAT-CR scale, the research team will also utilize clinical assessment of the potential participants to determine their capacity to consent to participating in the research, in a similar manner to determining capacity for clinical treatments as outlined in clinical guidelines (55). During the research consent conversation, the research team member will look for evidence of the same four components of decision-making capacity. Specifically, research team members will use their clinical expertise and judgement to check that participants: (a) have an understanding of what participating in this study entails and the associated benefits and risks; (b) have an appreciation for the how these benefits and risks apply to them specifically; (c) are able to show reasoning that their decision to participate aligns with their values; and (d) are able to

communicate their choice to the research team. If during the consent conversation, the person obtaining consent finds, based on their clinical judgement, that any of these criteria are not found to be met, consent will have to be obtained by from the substitute decision maker.

Scales to assess adverse events related to the study medication

UKU side effect rating scale: The UKU side effect rating scale is a short and easy-to-use instrument that captures the core dimensions of side effects in patients using psychotropic medications. The UKU side effect rating scale is widely used in both research and in clinical settings (57), and is delivered by interviewing and observing the patients and their caregivers. Herein, we will adopt the UKU side effect rating scale specifically adjusted to adults with IDD, which consists of 35 items out of the original 48 items. This revised checklist seems more feasible to observe items that are concrete and objective than items based on the patients' subjective experiences (68). Approximately 20 minutes to complete.

Scales to measure caregiver's stress and crisis

1. The Stress subscale of Depression Anxiety Stress Scales (DASS-21): The 7-item Stress subscale of the original DASS-21 (64) is used to assess perceived feelings of parent stress. Items measure feelings experienced over the previous week and are scored on a 4-point Likert scale from 0 ("did not apply to me at all") to 3 ("applied to me very much, or most of the time"), providing total scores between 0 and 21 for stress. <5 minutes to complete

2. Brief Family Distress Scale (BFDS): The BFDS (65) measures the subjective experience of crisis in families of people with neurodevelopmental disabilities. This measure is meant to assess the experience from the caregiver's perspective and is placed along a continuum of distress, from mild stress to qualitatively distinct and immediate periods of crisis. <1 minute to complete.

Scales to measure acceptability

Custom-designed acceptability questionnaire: This questionnaire is similar to that used in (31), which inquires the acceptability of all study components, including recruitment, withdrawal rate, study visit attendance, protocol adherence and the time burden of completing questionnaires.

Other measures

1. Demographic questionnaire
2. Current and Concomitant medications (including type, dose, and duration of treatment)[1]

---

[1]The high dose of benzodiazepine (more than the benzodiazepine equivalent to lorazepam 2 mg daily) and other cannabinoids, which are still used 4 weeks before the start of the use of the study drug (titration phase), are not

3. Height
4. Weight

	18b	All participants entering the dose titration phase will be included in the description of the safety profile and tolerability of nabilone (the Primary Objective). Participants completing the dose titration stage will be included in the final statistical analysis for the Exploratory Objective (i.e., exploring changes in SBPs in participants pre- to post-treatment), regardless of whether they complete the full protocol. This approach is consistent with the intention-to-treat principle.
Data management	19	Participant data will be de-identified and linked with study-specific identification numbers. The data will be recorded and stored online through Research Electronic Data Capture (REDCap), CAMH. Data and all appropriate documentation will be stored for a minimum of 25 years after the completion of the study.
Statistical methods	20a	A paired t-test will be used to investigate the pre- to post-treatment behavioral changes. Exploratory analyses will be performed to determine predictors of response including baseline individual features, using logistic regression. Safety profiles will be described by the percentage of each adverse effect. For MRI measures, non-parametric tests will be used to explore the brain changes with nabilone treatment, assuming that only a few participants will complete MRI scans. In addition, it is expected that the investigators will conduct further exploratory analyses (not specified here) on these data.
	20b	In addition, it is expected that the investigators will conduct further exploratory analyses (not specified here) on these data.
	20c	Any research information recorded for, or resulting from, participation in this research study prior to the date that the participant formally withdraws from the study will be retained and may continue to be used and disclosed by the investigators for research purposes. However, no new data will be further collected. There is no exception to the Safety Follow-up visit.<br><br>As the purpose of this phase I study is to investigate the tolerability of nabilone in adults with IDD, we will not recruit any new participants to replace the ones who withdraw from the entire trial
**Methods: Monitoring**		
Data monitoring	21a	We do not have an independent Data and Safety Monitoring Board (DSMB) in the current trial for the following reasons:

permitted. Other concurrent medications and non-medication treatments (including behavioural therapy, psychological therapy, occupational therapy, support group etc.) are permitted before and during the trial.

This proposal is a study that involves single data collection site (CAMH), unblinded data, short-term data collection, small numbers of participants (N = 30), and clear criteria regarding removing participants from the trial. This study of short-term intervention with small numbers of participants cannot generate enough data for interim analyses to permit the establishment of an effective study-wise stopping rule. Moreover, this proposal is the investigator-initiated trial and there are no biomedical or financial conflicts of interest regarding using Nabilone or any cannabinoids to treat individuals with IDD within the research team. Lastly, based on the published data, the possible adverse outcomes for participants in the current trial appears moderate (temporary, non-life-threatening conditions).

These rationales conform to Tri-Council Policy Statement: Ethical Conduct for Research Involving Humans – TCPS 2 (2018) – Chapter 11: Clinical Trials 11.7 (https://ethics.gc.ca/eng/tcps2-eptc2_2018_chapter11-chapitre11.html#7).

21b **Termination of the entire study**

Reasons for withdrawing individual participants from the study may include one or more of the following:

a) Failure to continue to meet the inclusion criteria.
b) Changes in participants' behaviours or situations, which meet the exclusion criteria. For example, participants become pregnant, newly use other cannabinoids, or newly use illegal psychomimetic substances as specified in Section 3.3, after the start of the trial.
c) Major protocol violation.
d) Participant lost to follow-up.
e) Withdrawal of consent: Participation in the study is voluntary, and the participant can refuse or stop participation at any time. The participant's refusal to participate or withdraw from the study will not interfere in any way with the relationship with his/her doctor or hospital.
f) Inability to tolerate the study medication or procedure.
g) Serious adverse effects related to the study medication.
h) New information shows that the study intervention is no longer in the participant's best interest.

In addition to these discontinuation criteria, any participant may be discontinued from the study at the discretion of the investigators if this is deemed to be in the best interest of the participant. The decision may be made either to protect the participant's health and safety, or because it is part of the research plan that people who develop certain conditions may not continue to participate. To ensure safety, we will arrange a Safety

		Follow-up visit, which is the same as the one for those who fully complete the trials, for participants withdrawn from the study.<br><br>Any research information recorded for, or resulting from, participation in this research study prior to the date that the participant formally withdraws from the study will be retained and may continue to be used and disclosed by the investigators for research purposes. However, no new data will be further collected. There is no exception to the Safety Follow-up visit.<br><br>As the purpose of this phase I study is to investigate the tolerability of nabilone in adults with IDD, we will not recruit any new participants to replace the ones who withdraw from the entire trial.
Harms	22	Adverse events (AEs) will be documented and reported as mandated by current regulations. AEs will be assessed at each study visit and telephone follow-up. All AEs, whether reported by the participant, SDM, or observed by study staff/investigators, will be recorded on the AE log along with a brief description, start date/resolution date and any action taken. The AE log will be initiated by the PI (Dr. Lin), who will make the determination on the relationship of the AE to the investigational drug/study procedures. All unexpected AEs determined by the PI to have a causal relationship with the investigational product or as a result of study procedures, will be reported to CAMH REB as part of the annual ethics approval renewal process. Where the event is deemed to meet CAMH REB reporting criteria, the PI will notify the REB. Every adverse event will be assessed and recorded in the participant's file by the PI or another physician involved in the study. The PI will report all Serious and Unexpected Adverse Drug Reactions (SUADR) to Health Canada as per Division 5 regulations.<br><br>If participants have AEs from participation in this study, medical care will be provided in the same way they would normally get medical care (for example, by going to their family doctor or seeking emergency medical treatment if needed). If participants withdraw from the study because they are unable to tolerate any AEs, or because of serious AEs, we will arrange a Safety Follow-up visit, which is the same as the one for those who fully complete the trials. If participants can tolerate the AEs, and the investigators consider that the continuation of participation in the study is still in the best interest of these participants, the frequency of the regular phone check-up during the open label phase will be increased from once weekly to once every two days to make sure these AEs do not progress to intolerable or more serious ones. In addition to intolerable AEs/severe AEs, there are other criteria which will remove participants from continuing the study.
Auditing	23	Internal audits will be led by CAMH's Quality Assurance team and led by a Study Monitor. The first will be held no longer than 1 month after the 1st

participant receives the first dose of investigational product (Nabilone). Following audits will take place no longer than 6 months following the previous audit or no longer than 1 month after the 10th, 20th, 30th participants receive their first dose of investigational product.
During these audits, the following will be reviewed:

1. All essential documents for this study (i.e. Investigator Study Binder)
2. Signed informed consent forms for all screened and enrolled participants
3. All CRF binders and source documentation associated with the data for this study
4. Adverse events, reports of protocol compliance
5. Study drug storage area access and accountability

Ethics and dissemination

Research ethics approval	24	This study has been approved by the CAMH Research Ethics Board (CAMH REB #135-2020) and Health Canada (Control #256517); has been registered on ClinicalTrials.gov (Identifier: NCT05273320).
Protocol amendments	25	Any modifications to the protocol which may impact on the conduct of the study, potential benefit of the patient or may affect patient safety, including changes of study objectives, study design, patient population, sample sizes, study procedures, or significant administrative aspects will require a formal amendment to the protocol. Such amendment will be agreed upon by the study team, and approved by the CAMH REB prior to implementation and notified to Health Canada in accordance with Canadian regulations. Administrative changes of the protocol are minor corrections and/or clarifications that have no effect on the way the study is to be conducted. These administrative changes will be agreed upon by study team, and will be documented in a memorandum. The CAMH REB may be notified of administrative changes at the discretion of study team.
Consent or assent	26a	**Consent**

- The research assistant will schedule the first appointment (research screening visit) to discuss the study and obtain written informed consent from the participant or their SDM.
  - The participant or their SDM will be provided with the informed consent form either by email, mail, or secure file transfer (as noted in the Telephone Contact Script) before the consent discussion.
- SDM consent is included as the patient may not have the capacity to make the decision about study participation. As per the Tri-Council Policy Statement: Ethical Conduct for Research Involving

Humans, principle of respect for persons implies that those who lack the capacity to make decisions for themselves should have the opportunity to participate in research that may be of benefit to themselves or others. SDMs may decide whether participation in a particular study would be appropriate. These principles involve considerations of concern for welfare and justice.

- Consent will be documented on the written Informed Consent Form (ICF). This will include signatures from the participant/SDM, the person conducting the consent discussion, and the witness (if applicable). Full explanation about this study will be provided to the participants and/or their SDMs.
    - If the consent discussion is conducted in person, the participant/SDM will be provided with a completed and signed copy of the informed consent form at the end of their visit.
- The MacArthur Competence Assessment Tool for Clinical Research (MacCAT-CR) will be applied to both of them as needed in order to ensure that they understand the information presented in the ICF. A score of 70% or higher in the MacCAT-CR will be required to pass.
    - 1) If the participant passes the MacCAT, written informed consent will be obtained from the participant.
    - 2) If the participant is not able to complete or does not pass the MacCAT and their SDM passes the MacCAT, written informed consent will be obtained from the SDM.
- If the participant and their SDM choose to have the screening assessment and consent discussion virtually implemented (an elective process), they will receive the informed consent form either by email, mail, or secure file transfer (as noted in the Telephone Contact Script) before the consent discussion. If the participant/SDM chooses to receive the informed consent form by mail, after virtually conducting the consent discussion, we will document remote consent by acquiring a written signature from the prospective participant or their SDM on the paper informed consent form and have it mailed back to the research team using a pre-paid postage envelope. The research team member who conducted the consent discussion would then sign this informed consent form. Alternately, if the participant/SDM chooses to receive the informed consent form by email or secure file transfer, the participant/SDM will print and sign the informed consent form, and mail, fax, scan or send a photograph of the completed form back to the study team. If participants/SDMs choose to return the signed informed consent form by mail, we will provide a pre-paid postage envelope.
- During videoconferencing/teleconferencing, we will assess capacity and address any questions raised by prospective participants/SDMs prior to documenting informed consent, as identical to a consent discussion conducted in person.

- The participant/SDM will receive a fully signed, complete copy of the informed consent form within 10 days following the informed consent process. This complete copy will be distributed by mail, email or CAMH secure file transfer based on the method the participant/SDM agreed to.
- The research procedures will only begin after the informed consent form signed by the participant/SDM is received and signed by the research team member who conducted the consent discussion. However, the research procedures may begin prior to the participant/SDM's receiving a copy of the fully signed, complete informed consent form.

	26b	N/A
Confidentiality	27	There is a potential risk of breach of confidentiality that is inherent in all research protocols. Breach of confidentiality will be minimized by the staff who will maintain research data (identified only by participant code number not related to name, or date of birth) in separate charts and a dedicated password protected electronic database. A list of participant names, their ID numbers, and information about how they can be reached will be kept in a separate locked cabinet with access only to study personnel authorized by the PI. Procedures have been established, and will be followed, to minimize the risk of breach of confidentiality. Procedures to maintain confidentially include: (1) formal training sessions for all research staff emphasizing the importance of confidentiality; (2) specific procedures developed to protect participants' confidentiality, and (3) formal mechanisms limiting access to information that can link data to individual participants. All information obtained from participants will be kept as confidential as possible. Computer based files/data will be entered into password-secured databases and paper-based files will be stored in a secure location. These data will only be accessible to personnel involved in the study, and they will abide by confidentiality regulations of the REB. The ethics committee will be granted direct access to the study participants' original medical records for verification of study procedures and/or data, without violating the confidentiality of the participants, to the extent permitted by the law and regulations.<br><br>The brain imaging information collected as part of this study may be shared on public scientific repositories (e.g. openfmri.org). In this case, study IDs associated with the MRI scans will be changed to a newly anonymized ID. Any identifying information in the scan data will be strictly stripped; for example any portion of the scans that might allow an image of the participant's face will be removed (so called "defacing"). In addition to the scans, basic demographic information (e.g. age and gender) and some summary clinical scores collected may be shared. This information will be shared in order to make the best possible scientific use of the information collected as part of this study, for example by allowing other researchers

		around the world conducting similar research to directly compare their results to what we collect at CAMH. This also allows for greater transparency in research, where other researchers can directly examine information collected across studies to ensure the results are correct. Data will not be added to repositories until the study is completed. This data will be anonymized and not contain any personal health information.<br><br>Participants will not be identified by name in any publication of research results. Results will be published as group data without the use of characteristics that would identify individual participants.
Declaration of interests	28	All authors have declared that they have no competing or potential conflict of interest or financial interests, which may arise from being named as an author on the manuscript or as the co-investigators of the trial.
Access to data	29	Principal Investigator and all co-investigator will be given access to the cleaned data sets. Project data sets will be housed on the CAMH RedCap site created for the study, and all data sets will be password protected. To ensure confidentiality, data dispersed to project team members will be blinded of any identifying participant information.
Ancillary and post-trial care	30	If participants have adverse events from participation in this study, medical care will be provided in the same way they would normally get medical care
Dissemination policy	31a	The study findings will be presented at scientific conferences and published in peer-reviewed journals. Only aggregated results with individual identifying information removed will be presented. We will also leverage the patient and family engagement resources within the Azrieli Adult Neurodevelopmental Centre, CAMH, and The Hospital for Sick Children (SickKids), Toronto to translate lessons learned from this study (e.g., for a robust recruitment plan for a larger trial) and explore how to use the information gleaned to build capacity to carry out these types of studies in an inclusive manner. Specifically, we will highlight and disseminate the key results from this Phase I study with the families from across Canada who are linked to the Azrieli Adult Neurodevelopmental Centre, through the Health Care Access, Research and Developmental Disabilities (H-CARDD) Program (https://www.porticonetwork.ca/web/hcardd), as well as the Province of Ontario Neurodevelopmental Network (POND; https://pond-network.ca), newsletters to relevant stakeholders, and presentations at community meetings. In sum, throughout this process, we will seek input from stakeholder advisors (i.e., self-advocates with IDD, caregivers and families, via our established participatory research framework at CAMH and SickKids). Findings will be integrated and discussed with stakeholder advisors in an effort to: (i) identify meaningful ways to disseminate research findings to health service providers and stakeholders, and (ii) plan a subsequent medication clinical trial for this population in a more inclusive manner.
	31b	Topics suggested for presentation or publication will be circulated to

		PI and coinvestigators. Substantive contributions to the design, conduct, interpretation, and reporting of a clinical trial are recognised through the granting of authorship on the conference abstract and final trial papers. Individuals who fulfil authorship criteria will not remain hidden and will have final authority over manuscript content. those who do not fulfil such criteria should not be granted authorship (guest authorship).
	31c	The study findings will be presented at scientific conferences and published in peer-reviewed journals. Only aggregated results with individual identifying information removed will be presented.
**Appendices**		
Informed consent materials	32	See attached Informed Consent Form, Participant Assent Form, and Acceptability Questionnaire
Biological specimens	33	N/A

*It is strongly recommended that this checklist be read in conjunction with the SPIRIT 2013 Explanation & Elaboration for important clarification on the items. Amendments to the protocol should be tracked and dated. The SPIRIT checklist is copyrighted by the SPIRIT Group under the Creative Commons "Attribution-NonCommercial-NoDerivs 3.0 Unported" license.

References

1. Morris SP, Fawcett G, Brisebois L, Hughes J. A demographic, employment and income profile of Canadians with disabilities aged 15 years and over, 2017: Statistics Canada= Statistque Canada; 2018.
2. McBride O, Heslop P, Glover G, Taggart L, Hanna-Trainor L, Shevlin M, et al. Prevalence estimation of intellectual disability using national administrative and household survey data: The importance of survey question specificity. International Journal of Population Data Science. 2021;6(1).
3. Cooper SA, van der Speck R. Epidemiology of mental ill health in adults with intellectual disabilities. Curr Opin Psychiatry. 2009;22(5):431-6.
4. Bowring DL, Totsika V, Hastings RP, Toogood S, Griffith GM. Challenging behaviours in adults with an intellectual disability: A total population study and exploration of risk indices. The British journal of clinical psychology. 2017;56(1):16-32.
5. de Winter CF, Jansen AA, Evenhuis HM. Physical conditions and challenging behaviour in people with intellectual disability: a systematic review. J Intellect Disabil Res. 2011;55(7):675-98.
6. Dawson F, Shanahan S, Fitzsimons E, O'Malley G, Mac Giollabhui N, Bramham J. The impact of caring for an adult with intellectual disability and psychiatric comorbidity on carer stress and psychological distress. J Intellect Disabil Res. 2016;60(6):553-63.
7. National Collaborating Centre for Mental H. National Institute for Health and Care Excellence: Clinical Guidelines. Challenging Behaviour and Learning Disabilities: Prevention and Interventions for People with Learning Disabilities Whose Behaviour Challenges. London: National Institute for Health and Care Excellence (UK)
Copyright © The British Psychological Society & The Royal College of Psychiatrists, 2015.; 2015.
8. Sullivan WF, Diepstra H, Heng J, Ally S, Bradley E, Casson I, et al. Primary care of adults with intellectual and developmental disabilities: 2018 Canadian consensus guidelines. Canadian family physician Medecin de famille canadien. 2018;64(4):254-79.
9. Lunsky Y, Khuu W, Tadrous M, Vigod S, Cobigo V, Gomes T. Antipsychotic Use With and Without Comorbid Psychiatric Diagnosis Among Adults with Intellectual and Developmental Disabilities. Canadian journal of psychiatry Revue canadienne de psychiatrie. 2018;63(6):361-9.
10. Lunsky Y, Modi M. Predictors of Psychotropic Polypharmacy Among Outpatients With Psychiatric Disorders and Intellectual Disability. Psychiatric services (Washington, DC). 2018;69(2):242-6.
11. Deutsch SI, Burket JA. Psychotropic medication use for adults and older adults with intellectual disability; selective review, recommendations and future directions. Progress in neuro-psychopharmacology & biological psychiatry. 2021;104:110017.
12. Vereenooghe L, Flynn S, Hastings RP, Adams D, Chauhan U, Cooper SA, et al. Interventions for mental health problems in children and adults with severe intellectual disabilities: a systematic review. BMJ Open. 2018;8(6):e021911.
13. Sheehan R, Horsfall L, Strydom A, Osborn D, Walters K, Hassiotis A. Movement side effects of antipsychotic drugs in adults with and without intellectual disability: UK population-based cohort study. BMJ Open. 2017;7(8):e017406.
14. Vancampfort D, Schuch F, Van Damme T, Firth J, Suetani S, Stubbs B, et al. Metabolic syndrome and its components in people with intellectual disability: a meta-analysis. J Intellect Disabil Res. 2020;64(10):804-15.
15. Kolla NJ, Mishra A. The Endocannabinoid System, Aggression, and the Violence of Synthetic Cannabinoid Use, Borderline Personality Disorder, Antisocial Personality Disorder, and Other Psychiatric Disorders. Frontiers in behavioral neuroscience. 2018;12:41.

16. Murillo-Rodriguez E, Pastrana-Trejo JC, Salas-Crisóstomo M, de-la-Cruz M. The Endocannabinoid System Modulating Levels of Consciousness, Emotions and Likely Dream Contents. CNS & neurological disorders drug targets. 2017;16(4):370-9.
17. Capuron L, Castanon N. Role of Inflammation in the Development of Neuropsychiatric Symptom Domains: Evidence and Mechanisms. Current topics in behavioral neurosciences. 2017;31:31-44.
18. Calsolaro V, Edison P. Neuroinflammation in Alzheimer's disease: Current evidence and future directions. Alzheimers Dement. 2016;12(6):719-32.
19. Krefft M, Frydecka D, Zalsman G, Krzystek-Korpacka M, Śmigiel R, Gębura K, et al. A pro-inflammatory phenotype is associated with behavioural traits in children with Prader-Willi syndrome. European child & adolescent psychiatry. 2020.
20. Marsland AL, Prather AA, Petersen KL, Cohen S, Manuck SB. Antagonistic characteristics are positively associated with inflammatory markers independently of trait negative emotionality. Brain Behav Immun. 2008;22(5):753-61.
21. Coccaro EF, Lee R, Coussons-Read M. Elevated plasma inflammatory markers in individuals with intermittent explosive disorder and correlation with aggression in humans. JAMA Psychiatry. 2014;71(2):158-65.
22. Hosie S, Malone DT, Liu S, Glass M, Adlard PA, Hannan AJ, et al. Altered Amygdala Excitation and CB1 Receptor Modulation of Aggressive Behavior in the Neuroligin-3(R451C) Mouse Model of Autism. Frontiers in cellular neuroscience. 2018;12:234.
23. Martin M, Ledent C, Parmentier M, Maldonado R, Valverde O. Involvement of CB1 cannabinoid receptors in emotional behaviour. Psychopharmacology (Berl). 2002;159(4):379-87.
24. Rodríguez-Arias M, Navarrete F, Blanco-Gandia MC, Arenas MC, Aguilar MA, Bartoll-Andrés A, et al. Role of CB2 receptors in social and aggressive behavior in male mice. Psychopharmacology (Berl). 2015;232(16):3019-31.
25. Bie B, Wu J, Foss JF, Naguib M. An overview of the cannabinoid type 2 receptor system and its therapeutic potential. Curr Opin Anaesthesiol. 2018;31(4):407-14.
26. Rodriguez-Arias M, Navarrete F, Daza-Losada M, Navarro D, Aguilar MA, Berbel P, et al. CB1 cannabinoid receptor-mediated aggressive behavior. Neuropharmacology. 2013;75:172-80.
27. Kirtley OJ, O'Carroll RE, O'Connor RC. The role of endogenous opioids in non-suicidal self-injurious behavior: methodological challenges. Neuroscience and biobehavioral reviews. 2015;48:186-9.
28. Bloomfield MAP, Hindocha C, Green SF, Wall MB, Lees R, Petrilli K, et al. The neuropsychopharmacology of cannabis: A review of human imaging studies. Pharmacol Ther. 2019;195:132-61.
29. Hasler G, Buchmann A, Haynes M, Müller ST, Ghisleni C, Brechbühl S, et al. Association between prefrontal glutamine levels and neuroticism determined using proton magnetic resonance spectroscopy. Translational psychiatry. 2019;9(1):170.
30. Hess P. Cannabis and autism, explained 2020 [Available from: https://www.spectrumnews.org/news/cannabis-and-autism-explained/.
31. Efron D, Taylor K, Payne JM, Freeman JL, Cranswick N, Mulraney M, et al. Does cannabidiol reduce severe behavioural problems in children with intellectual disability? Study protocol for a pilot single-site phase I/II randomised placebo controlled trial. BMJ Open. 2020;10(3):e034362.
32. Efron D, Freeman J. Medical cannabis for paediatric developmental-behavioural and psychiatric disorders. J Paediatr Child Health. 2018;54(7):715-7.
33. Abramovici H. Information for Health Care Professionals: Cannabis (marihuana, marijuana) and the cannabinoids. Ottawa: Health Canada, 2013. 2018.

34. Elsaid S, Kloiber S, Le Foll B. Effects of cannabidiol (CBD) in neuropsychiatric disorders: A review of pre-clinical and clinical findings. Progress in molecular biology and translational science. 2019;167:25-75.
35. Atakan Z. Cannabis, a complex plant: different compounds and different effects on individuals. Ther Adv Psychopharmacol. 2012;2(6):241-54.
36. CADTH Rapid Response Reports. Long-term Nabilone Use: A Review of the Clinical Effectiveness and Safety. Ottawa (ON): Canadian Agency for Drugs and Technologies in Health Copyright © 2015 Canadian Agency for Drugs and Technologies in Health.; 2015.
37. Herrmann N, Ruthirakuhan M, Gallagher D, Verhoeff N, Kiss A, Black SE, et al. Randomized Placebo-Controlled Trial of Nabilone for Agitation in Alzheimer's Disease. The American journal of geriatric psychiatry : official journal of the American Association for Geriatric Psychiatry. 2019;27(11):1161-73.
38. Hillen JB, Soulsby N, Alderman C, Caughey GE. Safety and effectiveness of cannabinoids for the treatment of neuropsychiatric symptoms in dementia: a systematic review. Therapeutic advances in drug safety. 2019;10:2042098619846993.
39. Black N, Stockings E, Campbell G, Tran LT, Zagic D, Hall WD, et al. Cannabinoids for the treatment of mental disorders and symptoms of mental disorders: a systematic review and meta-analysis. The lancet Psychiatry. 2019;6(12):995-1010.
40. Peball M, Krismer F, Knaus HG, Djamshidian A, Werkmann M, Carbone F, et al. Non-Motor Symptoms in Parkinson's Disease are Reduced by Nabilone. Annals of neurology. 2020.
41. Kruger T, Christophersen EJJoD, Pediatrics B. An open label study of the use of dronabinol (Marinol) in the management of treatment-resistant self-injurious behavior in 10 retarded adolescent patients. Cannabinoids. 2006;27(5):433.
42. Kurz R, Blaas KJC. Use of dronabinol (delta-9-THC) in autism: a prospective single-case-study with an early infantile autistic child. J Dev Behav Pediatr. 2010;5(4):4-6.
43. Bedi G, Cooper ZD, Haney M. Subjective, cognitive and cardiovascular dose-effect profile of nabilone and dronabinol in marijuana smokers. Addict Biol. 2013;18(5):872-81.
44. Elsohly MA, Gul W, Wanas AS, Radwan MM. Synthetic cannabinoids: analysis and metabolites. Life Sci. 2014;97(1):78-90.
45. van Amsterdam J, Brunt T, van den Brink W. The adverse health effects of synthetic cannabinoids with emphasis on psychosis-like effects. Journal of psychopharmacology (Oxford, England). 2015;29(3):254-63.
46. Cowling T, MacDougall D. CADTH Rapid Response Reports. Nabilone for the Treatment of Post-Traumatic Stress Disorder: A Review of Clinical Effectiveness and Guidelines. Ottawa (ON): Canadian Agency for Drugs and Technologies in Health
Copyright © 2019 Canadian Agency for Drugs and Technologies in Health.; 2019.
47. Teva Canada Limited. TEVA-NABILONE (Product Monograph). In: Teva Canada Limited, editor. Canada2012.
48. Devinsky O, Cross JH, Laux L, Marsh E, Miller I, Nabbout R, et al. Trial of Cannabidiol for Drug-Resistant Seizures in the Dravet Syndrome. N Engl J Med. 2017;376(21):2011-20.
49. Agarwal V, Sitholey P, Kumar S, Prasad M. Double-blind, placebo-controlled trial of clonidine in hyperactive children with mental retardation. Mental retardation. 2001;39(4):259-
50. Ware MA, St Arnaud-Trempe E. The abuse potential of the synthetic cannabinoid nabilone. Addiction (Abingdon, England). 2010;105(3):494-503.
51. Haney M, Cooper ZD, Bedi G, Vosburg SK, Comer SD, Foltin RW. Nabilone decreases marijuana withdrawal and a laboratory measure of marijuana relapse. Neuropsychopharmacology : official publication of the American College of Neuropsychopharmacology. 2013;38(8):1557-65.

52. Appelbaum PS, Grisso T. MacArthur competence assessment tool for clinical research (MacCAT-CR): professional resource press/professional resource exchange; 2001.
53. Moser DJ, Schultz SK, Arndt S, Benjamin ML, Fleming FW, Brems CS, et al. Capacity to provide informed consent for participation in schizophrenia and HIV research. The American journal of psychiatry. 2002;159(7):1201-7.
54. Hein IM, Troost PW, Lindeboom R, Benninga MA, Zwaan CM, van Goudoever JB, et al. Accuracy of the MacArthur competence assessment tool for clinical research (MacCAT-CR) for measuring children's competence to consent to clinical research. JAMA pediatrics. 2014;168(12):1147-53.
55. Barstow C, Shahan B, Roberts M. Evaluating Medical Decision-Making Capacity in Practice. American family physician. 2018;98(1):40-6.
56. Moss S, Ibbotson B, Prosser H, Goldberg D, Patel P, Simpson N. Validity of the PAS-ADD for detecting psychiatric symptoms in adults with learning disability (mental retardation). Social psychiatry and psychiatric epidemiology. 1997;32(6):344-54.
57. Kenworthy L, Case L, Harms MB, Martin A, Wallace GL. Adaptive behavior ratings correlate with symptomatology and IQ among individuals with high-functioning autism spectrum disorders. J Autism Dev Disord. 2010;40(4):416-23.
58. Busner J, Targum SD. The clinical global impressions scale: applying a research tool in clinical practice. Psychiatry (Edgmont (Pa : Township)). 2007;4(7):28-37.
59. Shields RH, Kaat AJ, McKenzie FJ, Drayton A, Sansone SM, Coleman J, et al. Validation of the NIH Toolbox Cognitive Battery in intellectual disability. Neurology. 2020;94(12):e1229-e40.
60. Aman MG, Singh NN, Stewart AW, Field CJ. Psychometric characteristics of the aberrant behavior checklist. Am J Ment Defic. 1985;89(5):492-502.
61. Aman MG, McDougle CJ, Scahill L, Handen B, Arnold LE, Johnson C, et al. Medication and parent training in children with pervasive developmental disorders and serious behavior problems: results from a randomized clinical trial. J Am Acad Child Adolesc Psychiatry. 2009;48(12):1143-54.
62. Oliver P, Crawford M, Rao B, Reece B, Tyrer PJJoARiID. Modified Overt Aggression Scale (MOAS) for people with intellectual disability and aggressive challenging behaviour: a reliability study. 2007;20(4):368-72.
63. Esbensen AJ, Rojahn J, Aman MG, Ruedrich S. Reliability and validity of an assessment instrument for anxiety, depression, and mood among individuals with mental retardation. J Autism Dev Disord. 2003;33(6):617-29.
64. Henry JD, Crawford JR. The short-form version of the Depression Anxiety Stress Scales (DASS-21): construct validity and normative data in a large non-clinical sample. The British journal of clinical psychology. 2005;44(Pt 2):227-39.
65. Weiss JA, Lunsky YJJoC, Studies F. The brief family distress scale: A measure of crisis in caregivers of individuals with autism spectrum disorders. 2011;20(4):521-8.
66. Derks O, Heinrich M, Brooks W, Sterkenburg P, McCarthy J, Underwood L, et al. The Social Communication Questionnaire for adults with intellectual disability: SCQ-AID. Autism Res. 2017;10(9):1481-90.
67. Oliver C, Richards C. Practitioner Review: Self-injurious behaviour in children with developmental delay. J Child Psychol Psychiatry. 2015;56(10):1042-54.
68. Tveter AL, Bakken TL, Bramness JG, Røssberg JIJAiMH, Disabilities I. Adjustment of the UKU Side Effect Rating Scale for adults with intellectual disabilities. A pilot study. 2014.
69. Andrade AK, Renda B, Murray JE. Cannabinoids, interoception, and anxiety. Pharmacol Biochem Behav. 2019;180:60-73.
70. Eley SEA, McKechanie AG, Campbell S, Stanfield AC. Facilitating individuals and families affected by fragile X syndrome to participate in medication trials. J Intellect Disabil Res. 2020.

71. Van Essen DC, Smith SM, Barch DM, Behrens TE, Yacoub E, Ugurbil K. The WU-Minn Human Connectome Project: an overview. Neuroimage. 2013;80:62-79.
72. Yeh CH, Tseng RY, Ni HC, Cocchi L, Chang JC, Hsu MY, et al. White matter microstructural and morphometric alterations in autism: implications for intellectual capabilities. Mol Autism. 2022;13(1):21.
73. Canadian ADHD Resource Alliance (CADDRA). Canadian ADHD Practice Guidelines. Fourth ed. Toronto, Ontario: CADDRA; 2018.
74. Dahlberg SE, Shapiro GI, Clark JW, Johnson BE. Evaluation of statistical designs in phase I expansion cohorts: the Dana-Farber/Harvard Cancer Center experience. J Natl Cancer Inst. 2014;106(7).
75. Dimian AF, Symons FJ. A systematic review of risk for the development and persistence of self-injurious behavior in intellectual and developmental disabilities. Clin Psychol Rev. 2022;94:102158.
76. Shepherd V. An under-represented and underserved population in trials: methodological, structural, and systemic barriers to the inclusion of adults lacking capacity to consent. Trials. 2020;21(1):445.
