# Supplementary material for: Nabilone treatment for severe behavioral problems in adults with intellectual and developmental disabilities: Protocol for a phase I open-label clinical trial
Source: PLoS One. 2023 Apr 12;18(4):e0282114. doi: 10.1371/journal.pone.0282114 (PMC10096227; doi:10.1371/journal.pone.0282114)
Supplement: S5 File — (PDF) [file pone.0282114.s005.pdf]

<u>STUDY PROTOCOL</u>
<u>(V3.0 – 20DEC2021)</u>

Study Title: **Phase I pre-pilot open-label clinical trial of nabilone for severe behavioural problems (aggression) in adults with intellectual and developmental disabilities (N-AND)**

Principal/Qualified Investigator: Hsiang-Yuan Lin, MD (CAMH)

Co-Investigators: Tarek K. Rajji, MD, FRCPC (CAMH)
Yona Lunsky, PhD (CAMH)
Pushpal Desarkar, MD (CAMH)
Meng-Chuan Lai, MD, PhD (CAMH)
Stephanie Ameis, MD, MSc, FRCPC (CAMH)
Wei Wang, PhD (CAMH)
Elia Abi-Jaoude MSc, MD, PhD, FRCPC (The Hospital For Sick Children)

Collaborators: Anupam Thakur, MD (CAMH)
Amanda Sawyer, MD, FRCPC (CAMH)
Ms. Jyll Simmons (CAMH)
Fa-Hsuan Lin, PhD (Sunnybrook Research Institute)

Source of funding: The CAMH AFP Innovation Fund; Department of Psychiatry Excellence Funds

Investigators' affiliation: Adult Neurodevelopmental and Geriatric Psychiatry Division &
Azrieli Adult Neurodevelopmental Centre,
Centre for Addiction and Mental Health,
Department of Psychiatry, University of Toronto

Sponsor: CAMH

CAMH REB Ref.: 135/2020

The signature below denotes confirmation that this research study will be conducted according to all stipulations of the protocol, and according to local legal and regulatory requirements and ICH GCP guidelines.

______________________________ ________________________
Signature Date
PI/QI
CAMH

1. BACKGROUND/RATIONALE/PRESENT STATE OF KNOWLEDGE

1.1. Overall Aim:

About one to three percent of adults in Canada have an intellectual and developmental disability (IDD), and 20-40% of them have severe behavioural problems (SBP), including self-injurious behaviour, disruptive behaviour, or aggression. SBP are common contributors to morbidity and reduced quality of life for adults with IDD and their families. Current medications for SBP show equivocal effectiveness and are associated with a high risk of side effects. Innovative and safe treatments are urgently needed to increase quality of life and reduce morbidity, caregiver burden, and health care costs. While preclinical studies, anecdotal reports, and uncontrolled studies link SBP with an endocannabinoid mechanism and suggest that medical cannabinoids may be an efficacious and safe alternative to current medications for SBP in adults with IDD, rigorous evidence is still in the very early stages. Unlike cannabidiol (CBD), a cannabinoid with elusive mechanisms and dose ranges, nabilone, a marketed synthetic cannabinoid, has a clearer mechanism, favourable safety profile, and more predictable dosing. Data of safety and efficacy regarding nabilone in this population has never been published. Parallelly, evaluating the effects of nabilone using MRI (especially fMRI and ^{1}H MRS, which measure brain function and metabolism, respectively) optionally in some participants has the potential to reveal mechanisms underpinning behavioural changes post-treatment, thereby facilitating the development of better designs in using cannabinoid for SBP in IDD. Thus, we propose to conduct this investigator-initiated Phase I pre-pilot open-label clinical trial.

Primary Aim 1: To collect preliminary data on the tolerability and safety profile of nabilone in adults with IDD.
Hypothesis 1: To evaluate tolerability, we hypothesize that 80% of participants will complete the study protocol.
Hypothesis 2: To evaluate safety, we hypothesize that nabilone will be satisfactorily tolerated and have a favourable safety profile in adults with IDD, as quantified by lower rates of adverse events (AEs) and serious adverse events (SAEs) than those reported in the literature of antipsychotics in IDD.
Exploratory Aim 2: We will explore changes in SBP, as well as brain changes on MRI, which is optional, in participants pre-and post-treatment.

The trial includes a screening visit for eligibility, a baseline visit followed by a dose titration phase, and then a 4-week open label phase at a stable dose. At the end of the open label phase, participants will undergo a termination visit, after which nabilone will be tapered off over 8 days. And then, 2 weeks later, participants will undergo a last follow-up visit to ensure safety.

Findings from this project will inform the design of a series of follow-up clinical trials, i.e., a next-stage Phase I/II pilot placebo-controlled randomized controlled trial (RCT) to test the feasibility of a larger trial, followed by a Phase II/III multicentre RCT. This planned Phase I study and the ensuing trials will address an evidence-practice gap in the use of cannabinoid to meet a pressing need for services, community and families.

1.2. The Need for More Research to Develop Innovative Treatment for SBP in Adults with IDD:

About 1-3% of adults in Canada have an intellectual and developmental disability (IDD) (1), and 20-40% of them have severe behavioural problems (SBP), including self-injurious behaviour, disruptive behaviour, or aggression (2), in the absence of a confirmed psychiatric diagnosis. These SBP are associated with emotion dysregulation, pain, and sleep problems (3). SBP are major contributors to morbidity, functional impairments, missed opportunities for learning, reduced quality of life, and burden on families, health, education and disability sectors (4).

SBP in adults with IDD are extremely difficult to treat. Although psychosocial interventions are generally considered first-line treatments per national and international guidance (5, 6), for a subgroup of people with IDD and SBP, these options are not effective. Antipsychotics and other psychotropic medications are thus prescribed for up to 70% of adults with IDD (7, 8), off-label, and with equivocal efficacy (9). Further, people with IDD, relative to those without IDD, are at a higher risk of side effects of psychotropic agents and less able to report side effects (10, 11). These side effects include weight gain, metabolic disturbances, neurological symptoms, and avoidable death (12). Finally, because of suboptimal responses to currently available medications, polypharmacy is commonly used to treat SBP in adults with IDD (8), potentially leading to more adverse events (9). Thus, innovative and safer interventions are pressingly needed for this population.

1.3. Endocannabinoid System and SBP

Stimulated by Δ9-tetrahydrocannabinol (THC), the primary psychoactive ingredient in cannabis, cannabinoid receptors 1 (CB1) of the endocannabinoid system modulate the activity of serotonergic, noradrenergic, dopaminergic, GABAergic, and glutamatergic neurotransmitters and is a potent modulator of myriads of cognitive and behavioural processes including learning and attention, emotion regulation, sleep structure, and pain perception (13). Cannabinoid receptors 2 (CB2), also activated by THC, are mainly localized in peripheral immune cells and are expressed in the brain during neuroinflammation. Inflammatory processes alter the metabolism and functions of neurotransmitters, neuroendocrine activity and neural plasticity, leading to the development of neuropsychiatric symptoms (14). Pro-inflammatory markers are positively linked with aggression in children with Prader-Willi Syndrome (60% of them have IDD and those without IDD have some learning disabilities) (15). Neuroinflammation is modulated by CB2 agonist (16).

CB1/2 agonists have also been tested in preclinical studies and, at lower doses, have been shown to reduce aggression. Further, CB1/2 knockout mice show more aggression than wild mice (17, 18). A theoretical review also suggest CB1 modulation may be a future candidate for treatment of self-injury (19).

Human functional MRI (fMRI) data suggest that at low doses, THC attenuates anxiety responses and also reduces amygdala reactivity and its functional coupling with frontal regions while processing stimuli with a negative emotional content. Conversely, at higher doses, the effect of acute administration of THC becomes anxiogenic (20). Parallelly, human proton magnetic resonance spectroscopy (^{1}H MRS) studies indicate that the GABA and glutamate–glutamine balance, which is modulated by CB1, links with emotion regulation and related psychopathology (21).

1.4. Rationale for the Use of Nabilone in People with IDD and SBP

Anecdotal reports (22) and preliminary research (23) suggest that medicinal cannabinoid may be useful to treat SBP in youth with developmental disabilities. However, and despite high hopes

held by families, the scientific evidence is in very early stages. Most studies to date focused on cannabidiol (CBD), as THC use is associated with cognitive decrements, psychotic symptoms, and addictive in recreational marijuana users (24). However, the full mechanism and dose range of CBD, which has very low affinity for CB1/2, remains elusive. Prior evidence suggests limited beneficial properties of CBD for aggression and risk factors associated with SBP (25).

Nabilone is a synthetic oral THC analogue that acts as a partial agonist on both CB1/2 in humans. Thus, it mimics the effect of THC on aggression/emotion regulation, sleep, and pain, but with more predictable side effects, better safety profiles, and less euphoria (26). Clinical studies suggest that nabilone may be useful in alleviating agitation in dementia (27, 28), nightmares in post-traumatic stress disorder (PTSD) (29), non-motor symptoms (mood dysregulation, sleep, pain) in Parkinson's disease (30), and core symptoms in anxiety and pain disorders (29). Preliminary studies suggest that another synthetic THC, dronabinol (a full agonist at CB 1/2), may be effective in alleviating SBP in youth with IDD (31, 32). However, dronabinol has been withdrawn from the Canadian market for unknown reasons. Moreover, nabilone, relative to dronabinol, has better bioavailability (33), may have less adverse mental effects (because of partial vs. full agonist properties), and may be more effective in alleviating SBP (27).

Safety: Nabilone is indicated for severe nausea and vomiting associated with cancer chemotherapy and is commercially available in Canada. Its use is usually safe and satisfactorily tolerated. The most frequently reported adverse reactions to nabilone in previous clinical trials are as follows: drowsiness (66.0%), vertigo (58.8%), psychological high (38.8%), dry mouth (21.6%), depression (14.0%), ataxia (12.8%), blurred vision (12.8%), sensation disturbance (12.4%), anorexia (7.6%), asthenia (7.6%), headache (7.2%), orthostatic hypotension (5.2%), euphoria (4.0%) and hallucinations (2.0%) (26-28, 30, 34, 35). The safety profile and rate of AEs for nabilone are comparable to those reported in studies of CBD (36) and clonidine (37), and substantially more favourable than what has been reported for antipsychotics (9, 11, 12).

The addictive potential of nabilone is very low and unlikely (38), because it is associated with less euphoria, slower onset of action, and more difficult titration compared to smoking cannabis. Nabilone has been shown to help with sustained abstinence of marijuana (39). The incidence of nabilone-induced psychotic symptoms is rare, and was only reported in people with personal/family history of psychosis.

Taken together, the evidence suggests that nabilone is a promising treatment for SBP in adults with IDD. However, the efficacy and safety of nabilone in this population have yet to be studied.

Evaluating the effects of this novel nabilone intervention using MRI, especially fMRI and ^{1}H MRS, which measure brain function and metabolism, respectively, has the potential to reveal mechanisms underpinning behavioural changes post-treatment. This pharmacoimaging approach also enables the better understanding of response heterogeneity, thereby facilitating the development of better designs in using cannabinoid for SBP in IDD.

2. STUDY OBJECTIVES

To address the current state of evidence for nabilone use to treat SBP in adults with IDD, we propose to conduct the first Phase I pre-pilot open-label clinical trial with the following aims and hypotheses:

Primary Objective: To collect preliminary data on the tolerability and safety profile of nabilone in adults with IDD.
<u>Hypothesis 1:</u> To evaluate tolerability, we hypothesize that 80% of participants will complete the study protocol.
<u>Hypothesis 2:</u> To evaluate safety, we hypothesize that nabilone will be satisfactorily tolerated and have a favourable safety profile in adults with IDD, as quantified by lower rates of AEs than those reported in the literature of antipsychotics in IDD.

Exploratory Objective: We will explore changes in SBP, as well as brain changes on MRI, which is optional, in participants pre- and post-treatment.

<u>Significance/Innovation:</u>
This is the first study to investigate nabilone for SBP in people with IDD. We will conduct a Phase I open-label trial to assess nabilone safety in this population, but with a fixed dosage range since it is an already marketed product (40). Findings from this project will inform the design of a series of clinical trials led by the PI in the future, i.e., a next-stage Phase I/II pilot placebo-controlled RCT to test the feasibility of a large-scale RCT, followed by a Phase II/III multicentre RCT to test available options for this difficult-to-treat clinical problem (Fig. 1). The planned trial will address an evidence-practice gap in the use of cannabinoid to meet a pressing need for services, the community and families, which has been identified as a priority research area by adults with neurodevelopmental disabilities, family members and clinicians in an Ontario-wide priority setting exercise (rb.gy/thr2od).

<u>3. RESEARCH DESIGN/METHODS/ANALYSIS</u>

3.1. Study Design
This study is a Phase I pre-pilot open-label trial of nabilone in adults with IDD and SBP. As shown in Figure 1, the trial first includes a screening visit (S-V) for eligibility, then a baseline visit (V 0), followed by a dose titration phase, and then a 4-week open label phase at a stable dose. At the end of the open label phase, participants will undergo a termination visit (V 1), after which nabilone will be tapered off over 8 days. And then, 2 weeks later, participants will undergo a last follow-up visit (V-F) to ensure safety.

At S-V, and before the informed consent process, the participant will be assessed for capacity to provide informed consent. If they cannot pass the competence test using the MacArthur Competence Assessment Tool for Clinical Research (MacCAT-CR), then the written informed consent will be obtained from their substitute decision maker after seeking participants' assent.

At S-V, participants will also be assessed for eligibility based on the inclusion and exclusion criteria, including SBP defined as a score ≥18 on the Aberrant Behaviour Checklist-Irritability

subscale (ABC-I) and a score ≥4 on the Clinical Global Impressions-Severity scale (41). The SBP should present with a consistent pattern with the frequency of ≥1 time per week for >3 months.

If it is deemed necessary, research staff may review participants' medical charts to obtain clinically relevant information and/or additional information to confirm their eligibility. The investigator or research staff may also request information from the participant's physician.

At V 0 the following assessments will be completed:

- Clinical Global Impression – Severity scale (CGI-S) will be rated by the investigator to evaluate the severity of the behavioural problems.
- Measuring baseline vital signs and weight to monitor any AEs of nabilone.
- Assessment of participants' cognitive capacity using the NIH Toolbox® Cognition Battery. Previous evidence suggests that nabilone may cause acute mild decrement in attention and working memory (27). We will use the NIH Toolbox® Cognition Battery, which has been shown as a good candidate outcome measure to examine broad nonverbal cognitive changes for individuals with IDD (valid and reliable for those with a mental age of ≥5 years; implementable for those with a mental age of ≥3 years) (42), to assess the baseline attention and working memory.
- Assessment of SBP of participants, including behavioural problems using the ABC-I and Modified Overt Aggression Scale (MOAS), as reported by the caregivers. These scales will be the principal measures for detection of changes in SBP.
- Assessment of anxiety using the parent-rated General Anxiety Subscale of the Anxiety, Depression, and Mood Scale (ADAMS). Nabilone and THC at low doses have been shown to alleviate anxiety, thus this measure is used for exploratory aims to detect a signal of behavioural/mood change with nabilone use.
- Assessment of caregiver's stress and crisis using the Stress Subscale of Depression Anxiety Stress Scales (DASS-21) and Brief Family Distress Scale (BFDS). As SBP cause major caregiver burden, we aim to assess whether the caregiver's stress and distress would improve if nabilone also improves participants' SBP.
- Exploratory assessment of autism symptoms using the Social Communication Questionnaire for Adults with Intellectual Disability (SCQ-AID). Prior evidence suggests that autistic symptoms may be associated with the presentation of SBP (43). We aim to explore whether the baseline autistic symptoms would modulate treatment effects of nabilone.
- Optional assessment of MRI Imaging (dependent on the participant's ability to comply with the following assessments, in the opinion of the carers/parents and investigator).

After V 0, eligible participants will receive open-label nabilone starting with a dosage of 0.25 mg before sleep. During the dose titration phase (which could last up to 15 days), nabilone will be titrated in 0.25 mg increments every two days (with the dosing schedule "twice daily") after consultation with the study team during regular phone calls. This proposed up-titration schedule was decided based on a clinical trial of using Nabilone to treat non-motor symptoms in adults with Parkinson's disease (30). These regular phone calls will be carried out every two days during the whole dose titration phase until the participant reaches a stable dose for the open-label phase. The study team will also check whether the participants show any physical or mental changes which are believed to be attributed to AEs (based on the UKU side effect rating scale), and advise the participant and their caregivers on the next step of titration accordingly. Dose adjustments are

performed until the participant reaches the maximum permitted dose of 1 mg twice daily or experience intolerable AEs believed to be related to nabilone. If intolerable, the participant will use nabilone at the previous lower dose, entering the open label phase. Note that the suggested maximum nabilone daily dose per the product monograph is 6 mg. However, considering the vulnerability of the current target population, we choose to administer 2 mg/day at most in the current proposal following the maximum dose used in clinical trials in people with dementia (27) and Parkinson's disease (30). Moreover, nabilone is a THC analog; THC appears to reduce anxiety at low doses but becomes anxiogenic at high doses. Thus, administering nabilone at lower doses is theoretically consistent with the treatment objective for SBP.

Participants will be then on a stable, optimized nabilone dosage for four weeks, and then the trial ends with an on-site termination visit (V1). During the open-label trial phase, the study team will call the participant and their caregivers once weekly to perform a basic check-up, and to follow up with any questions or concerns from the trial processes. If there are any changes suspected to be associated with AEs, the UKU side effect rating scale will be assessed during the telephone check-up.

At V 1, the following post-treatment assessments will be completed:

- Assessment of adverse events using the UKU side effect rating scale, examined by the researcher or investigator, to systemically investigate the physical AEs of nabilone.
- Re-measuring of vital signs (including blood pressure to check orthostatic hypotension) and weight to monitor the physical AEs of nabilone.
- Re-assessment of participants' cognitive capacity using the NIH Toolbox® Cognition Battery, to monitor any acute cognitive change (AEs) due to nabilone.
- Re-assessment of caregiver rated ABC-I, MOAS, and General Anxiety Subscale of ADAMS. CGI-S and CGI-I will be rated by the investigator to evaluate the severity and improvement of the behavioural problems after nabilone treatment. These re-assessments are implemented to fulfil the exploratory study aim of assessing behavioural changes pre- and post-treatment of nabilone.
- Re-assessment of caregiver's stress and crisis using the Stress Subscale of DASS-21 and BFDS, to investigate changes in their stress and distress after participants takes nabilone.
- Optional re-assessment of MRI Imaging for those who finish the pre-treatment MRI and are willing to receive the second wave of MRI imaging. This is to investigate the change in brain function and structure pre- and post-treatment of nabilone.

Nabilone will be tapered in 0.25 mg daily decrements to prevent acute withdrawal effects. This This proposed down-titration schedule was decided based on a conservative modification from a clinical trial of using Nabilone to treat behavioural and psychological symptoms of dementia (27). Phone calls will be held every other day during the dose-tapering phase, to check whether there are any changes which are believed to be attributed to withdrawal effects. A safety follow-up visit (S-F) will be scheduled after 2 weeks of full discontinuation from the study drug.

The study team will pick up the blister pack from Research Pharmacy, and then distribute to the participant/caregiver. Upon receipt of the nabilone supplies, an inventory will be performed and a receipt log filled out and signed by the research staff who picks up the blister pack. Designated research staff/pharmacy must count and verify that the pickup contains all the items noted in the

shipment inventory. Any damaged or unusable nabilone in a given pick-up will be documented in the clinical trial files. Regarding dispensing the study medication, participants/caregivers will come to CAMH at 3 separate times to pick up nabilone (Table 1), including 1) to pick up the package for the whole dose titration phase and the first 6 days of the stable dose phase at the Pre-treatment Visit (V 0), 2) to pick up the package for the remaining days of the stable phase, and 3) to pick up the package for the dose tapering phase at the Post-treatment Visit (V 1). Nabilone will be prepared for each visit by Research Pharmacy Service. Participants/caregivers will be requested to document the details of using nabilone and other concomitant medications in the drug diary. They will also be asked and expected to return the empty or any leftover nabilone back to the study team, to help with drug accountability.

Regular study intervention reconciliation will be performed to document nabilone assigned, consumed, and remaining. This reconciliation will be logged on a drug accountability log, and signed and dated by delegated research and/or pharmacy staff.

At the completion of the clinical trial, there will be a final reconciliation of the nabilone dispensed, consumed and remaining, based on the drug diary, the remaining drugs which are returned. This reconciliation will be logged on an accountability form, and signed and dated by delegated research and/or pharmacy staff. Any discrepancies noted will be investigated, resolved, and documented prior to return or destruction of unused study intervention. Intervention destroyed on site will be documented in the clinical trial's files.

All participants entering the dose titration phase will be included in the description of the safety profile and tolerability of nabilone (the Primary Objective). Participants completing the dose titration stage will be included in the final statistical analysis for the Exploratory Objective (i.e., explore changes in SBP in participants pre-and post-treatment), regardless of whether they complete the full protocol. This approach is consistent with the intention-to-treat principle.

Figure 1. A schematic diagram of the study procedures, including all study visits and timelines. Abbreviation: S-V: a screening visit; V 0: a baseline visit; V 1: a visit at the end of open label phase; V-F: a follow-up visit to ensure safety; d: days; wk: weeks; FU: follow-up; max: maximum.

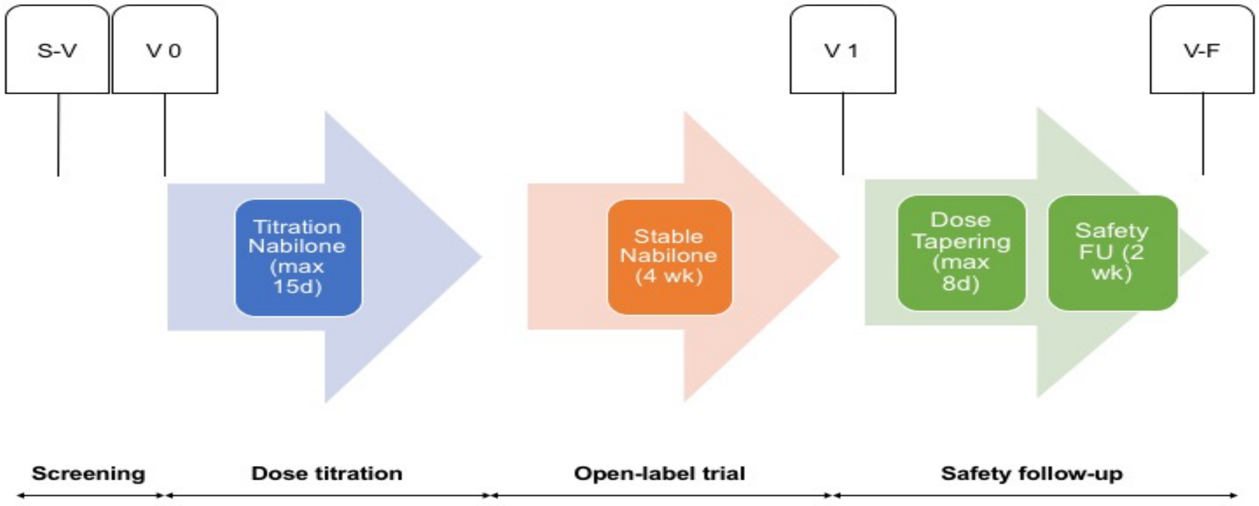

Table 1. Schedule of study visit procedures and assessments

	**Screening (S-V)**	**Pre-Treatment (V 0)**	**Titration**	**Open-label**	**Post-Treatment (V 1)**	**Safety Follow-up (V-F)**	**Visit option**
**Informed Consent**	•						
**Demographics**							
Dx of IDD and comorbidities (Moss-PAS (ID))	•						Either or[a]
Age	•						Either or
Sex/Gender	•						Either or
Height	•						In person
Weight	•	•			•	•	In person
Competence (MacCAT-CR)	•						Either or
Concomitant medication	•	•	•	•	•	•	Either or
Adaptive function (ABAS-3)	•						Either or
Compliance					•		Either or
**Safety Outcome**							
PE, Vital signs	•	•			•	•	In-person
Pregnancy	•						In-person
AEs (UKU)			•	•	•	•	Either or
NIH-Toolbox		•			•	•	In-person
**Clinical Effect Outcome**							
Primary							
ABC-I	•	•			•		Either or
Secondary							
MOAS		•			•		Either or
Anxiety-ADAMS		•			•		Either or
Stress-DASS-21		•			•		Either or
BFDS		•			•		Either or
CGI-S	•				•		Either or
CGI-I		•			•		Either or
Exploratory							
SCQ-AIQ		•					Either or
MRI (optional)		•			•		In-person

**Nabilone Dispense**							
Pick-up		•		•	•		In-person
Return (if applicable)				•	•	•	In-person
**Acceptability Evaluation**						•	Either or

Abbreviation: Dx = diagnosis; MacCAT-CR = MacArthur Competence Assessment Tool for Clinical Research; Moss-PAS (ID) = Moss Psychiatric Assessment Schedules; ABAS-3 = Adaptive Behavior Assessment System, Third Edition; PE = physical examination; AE = adverse event and severe adverse event; NIH-Toolbox = NIH Toolbox® Cognition Battery; ABC-I = Aberrant Behavior Checklist-Irritability Subscale; MOAS = Modified Overt Aggression Scale; CGI-I = Clinical Global Impressions (CGI) scale-Improvement; CGI-S = Clinical Global Impressions (CGI) scale-Severity; Anxiety-ADAMS = General Anxiety Subscale of Anxiety, Depression, and Mood Scale; Stress-DASS-21 = Stress Subscale of the short-form version of Depression Anxiety Stress Scales; BFDS = Brief Family Distress Scale; SCQ-AIQ = Social Communication Questionnaire for Adults with Intellectual Disability.

[a]: Either in-person or virtual visit

3.2. Assessments and visits (Table 1):

Screening assessments for participants and their caregivers include:

- Assessment of prior medical records to collect information of previous cognitive assessment regarding IQ, status of Disability of Ontario Services (DSO), co-occurring physical and psychiatric diagnoses, medication use and potential changes.
- Assessment of capacity to make an informed consent based on both the MacArthur Competence Assessment Tool for Clinical Research (MacCAT-CR) (44) and clinical assessment (45). This will be carried out before the informed consent process and formal assessment.
- Interview of the Moss Psychiatric Assessment Schedules (Moss-PAS (ID)) to the caregiver to assess for co-occurring mental health conditions.
- The Adaptive Behavior Assessment System, Third Edition (ABAS-3), Adult Form, will measure adaptive behaviour and function.
- Measuring of vital signs, weight and height.
- Urine pregnancy test for sexually active women of child-bearing potential.
- Caregiver rated Aberrant Behavior Checklist-Irritability subscale (ABC-I) will be used to define the SBP. Clinical Global Impression – Severity scale (CGI-S) will be rated by the investigator to evaluate the severity of the behavioural problems.

Baseline (pre-treatment) assessments include:

- Measuring of vital signs and weight.
- Assessment of participants' cognitive capacity using the NIH Toolbox® Cognition Battery.
- Assessment of SBP of participants using the ABC-I and MOAS, and anxiety using the General Anxiety Subscale of the ADAMS, as reported by the caregivers.
- Assessment of caregiver's stress and crisis using the Stress Subscale of DASS-21 and BFDS.
- Exploratory assessment of autism symptoms using the Social Communication Questionnaire for Adults with Intellectual Disability (SCQ-AID).

- Optional assessment of MRI Imaging (dependent on the participant's ability to comply with the following assessments, in the opinion of the carers/parents and investigator).
- Concomitant medication

Post-treatment assessments include:

- Assessment of adverse events using the UKU side effect rating scale, examined by the researcher or investigator.
- Re-measuring of vital signs and weight.
- Re-assessment of participants' cognitive capacity using the NIH Toolbox® Cognition Battery.
- Re-assessment of caregiver rated ABC-I, MOAS, General Anxiety Subscale of ADAMS. CGI-S and CGI-I will be rated by the investigator to evaluate the severity and improvement of the behavioural problems after nabilone treatment.
- Re-assessment of caregiver's stress and crisis using the Stress Subscale of DASS-21 and BFDS.
- Optional re-assessment of MRI Imaging for those who finish the pre-treatment MRI and are willing to receive the second wave of MRI imaging.
- Concomitant medication

Safety follow-up assessments include:

- Re-assessment of adverse events using the UKU side effect rating scale, examined by the researcher or investigator.
- Re-measuring of vital signs and weight.
- Concomitant medication
- Re-assessment of participants' cognitive capacity using the NIH Toolbox® Cognition Battery.
- Assessment of caregivers' acceptability of the study protocol using the custom-designed questionnaire, as completed by the caregiver.

Elective Virtual Assessment

To reduce barriers associated with traffic and time and to future-proof the research protocol in case of another COVID lockdown, we will provide the option for virtual assessments. As shown in the last column of Table 1, interviews that collect data on demographics, clinical history, diagnoses alongside medication use, competence, and adverse effects can be conducted virtually using teleconferencing/videoconferencing software (Webex). Additionally, a blank paper copy of outcome measures rated by caregivers (including the ABC-I, MOAS, Anxiety-ADAMS, Stress-DASS-21, BFDS, and SCQ-AIQ) can be electively sent to them via mail. Completed questionnaires can be mailed, faxed, scanned, or photographed back to the study team. If the caregivers choose to send the completed questionnaire by mail, we will provide a pre-paid postage envelope.

The virtual sessions will be undertaken in coordination with in-person sessions based on the same research standards. The participants and caregivers can flexibly choose either the in-person or virtual assessments for those elective options (Table 1). We will notify both the participant and their caregiver of this virtual option when explaining the study (based on the Telephone Contact Script) and discussing the informed consent.

If the participant/SDM/caregiver chooses to have a virtual informed consent process (as detailed in Section 5) and virtual screening assessment, we will only start the assessment after we receive

the signed informed consent form (with wet signatures). To reduce the barriers by enhancing the convenience and flexibility of participants/caregivers, if they choose to have screening assessments done virtually, the measures of the Screening Visit which require in-person assessments (i.e., pregnancy test, height, vital signs and physical examination) will be carried out in combination with those implemented during the pre-treatment assessment (V 0).

During virtual visits, the study team will document the records of virtual assessment on a study computer and sign the documents with e-signature first when working remotely. Then the person(s) who implements the virtual assessment will then print out the e-signed records and add a wet signature to verify the sources at first available opportunity on-site.

3.3 Selection Criteria

<u>Inclusion Criteria</u>
Participants of any sex or gender, race or ethnicity meeting all criteria listed below will be included in the study:

1) Aged ≥25 years, as medical cannabis should not be used in any person aged <25 as suggested by Health Canada (24).
2) Adults with a DSM-5 diagnosis of ID meeting: a. Full scale IQ <70 on a standardized cognitive assessment reported in their prior medical record; b. A deficit in adaptive function in at least one activity of life, as estimated by the Adaptive Behavior Assessment System, rated by the caregiver. For those whose verified records are not available, they are deemed eligible if they are connected with Disability Services Ontario. People with ID and other developmental disabilities, e.g., autism, Down syndrome, genetic conditions such as Angelman syndrome, fragile X syndrome, Prader-Willi Syndrome, etc., will also be enrolled.
3) SBP, including aggressive, disruptive, and/or self-injurious behaviours in any situation (home, day program, clinic, etc.), defined as a score ≥18 on the Aberrant Behaviour Checklist-Irritability subscale (ABC-I), and a score ≥4 on the Clinical Global Impressions-Severity scale (41). A consistent pattern of frequent SBP should occur for >3 months ≥1 time per week.
4) Sexually active women of child-bearing potential must have a negative urine pregnancy test at the screening visit.
5) Sexually active women of child-bearing must use an effective method of birth control at least from the start of last two normal menses before the screening visit to one month after the end of the study (completion of the safety visit). The accepted methods of contraception include total sexual abstinence, if it is the usual and preferred lifestyle, or consistently and correctly taking the oral hormonal contraceptive.
6) Adults who receive a blood test in recent 12 months, which shows liver function test with the ALT ≤3 times the upper limit of normal and bilirubin ≤2 times the upper limit of normal.
7) At least one month that needs to pass from the participation in another investigational drug trial to a given adults being allowed to participate in this trial.

<u>Exclusion Criteria</u>

1) History of hypersensitivity to any cannabinoid.
2) The presence of an unstable seizure disorder as defined by having not been seizure-free for at least 6 months or anticonvulsant treatment has not been stable for at least 4 weeks.

3) The presence of any clinically significant or unstable medical conditions, including cardiovascular, liver, kidney, pulmonary disease, presence of known congenital brain malformation, as per investigator assessment based on medical history and chart review.
4) The presence of a lifetime diagnosis of psychotic disorders, bipolar disorder, or substance use disorder, or current diagnosis of major depressive disorder or dementia, based on past psychiatric history noted in the medical chart, as well as Moss-PAS (ID) at S-V.
5) Family history of psychotic disorders.
6) Change in psychotropic medications less than 4 weeks prior to study drug use.
7) At the time of screening, each adult's medication list will be checked for drugs that are known to cause interactions with nabilone. When a given adult is taking any drugs or is taking a given medication exceeding a given dose) in the following list, he/she/they will be excluded.
    a. Currently on benzodiazepines at the dose more than the benzodiazepine equivalent to lorazepam 2 mg daily.
    b. Currently on medical psychostimulant, including methylphenidate (100 mg daily), lisdexamfetamine (70 mg daily), amphetamine/dextroamphetamine (Adderall XR®, 50 mg daily), dextroamphetamine (Dexedrine®, 50 mg daily) at the dose exceeding their respective maximum doses (as shown in the bracket after each agent) to treat ADHD in adults, based on the CADDRA guideline, www.caddra.ca.
    c. Currently on nonbenzodiazepine hypnotics, including zaleplon (10 mg daily), zolpidem (10 mg daily), and zopiclone (7.5 mg daily), at the dose exceeding their respective suggested safety doses (as shown in the bracket after each agent), based on Canadian Recalls and Safety Alerts (https://healthycanadians.gc.ca/recall-alert-rappel-avis/).
    d. Currently on any opioids.
    e. Currently on barbiturates.
    f. Drinking any alcohol one week before the screening visit.
    g. Recreational use of any psychomimetic drugs, including Ketamine, LSD, MDMA, Magic mushrooms, PCP, Salvia, GHB, Bath salts, Methamphetamine; the last use happens within one week before the screening visit.
8) Adults currently taking other cannabinoids, such as CBD or medical cannabis, from another source, unless participants and/or their caregivers are willing to stop this treatment for at least 4 weeks prior to entering the study.
9) Adults who might travel out of the area for a significant time during the study.
10) Adults who recently are participating in another investigational drug trial.
11) Pregnancy.
12) Sexually active women of child-bearing potential intended to give breastfeeding or to get pregnant.

3.4 Assessment/Scales/Measures

<u>Assessment for Diagnosis</u>

The Moss Psychiatric Assessment Schedules (The Moss-PAS (ID)): The Moss-PAS (ID) (previously called Mini PAS-ADD) (46) provides a wide-spectrum mental health assessment primarily designed for people with intellectual disabilities who have limited language or reduced cognitive development. This semi-structured diagnostic interview, delivered by the PI, is fully compliant with DSM-5 and ICD-11. It consists of 86 items on a 4-point scale: 0 (symptom not present)—3 (symptom is severe). The interview is divided into seven subscales: Depression,

Anxiety, Obsessive Compulsive Disorder, Mania, Psychosis, Unspecified disorder, and Autism. Approximately 30 min to complete.

Scales to measure autistic symptoms

Social Communication Questionnaire for Adults with Intellectual Disability (SCQ-AID) (47): The SCQ is a caregiver-rated dimensional measure of ASD symptom severity. The SCQ-AID is an adaptive version to improve the utility of the SCQ for adults with IDD. This newly developed algorithm has 24 items compared with the 40 items in the original instrument. <15 minutes to complete.

Scales to measure cognitive function

NIH Toolbox® Cognition Battery (NIHTB-CB): The NIHTB-CB is an iPad-based battery of brief memory, executive function, processing speed, and language tests, which was developed within the NIH Blueprint for Neuroscience Research. The NIHTB-CB has the potential to provide a highly standardized, objective, and scalable tool for use across laboratories and clinical trial sites and has been shown as a good candidate outcome measure to examine broad nonverbal cognitive changes for individuals with IDD (valid and reliable for those with a mental age of ≥5 years; implementable for those with a mental age of ≥3 years) (42). The NIHTB-CB will be used to monitor the change in cognitive performance pre-and post-treatment of nabilone. To allow for accurate scoring based on the established norm, the administration of NIHTB-CB will collect a set of limited personal identification information, including sex, age, and education level, plus study ID. This assessment will be delivered on-site at pre-treatment (V 0), post-treatment (V 1), and safety follow-up (V-F). It takes approximately 60 minutes to complete each full assessment. It should be noted that this administration time is approximate considering the different levels of motivation and cooperativeness in participants. The norming version for neurotypical people aged 3-85 years is intended to be administered with a 30-minute duration (48).

Scale to measure adaptive function

Adaptive Behavior Assessment System-III (ABAS-3), Adult Form. The ABAS-3 is a measure of adaptive behaviour that has been validated in a number of psychiatric populations and has been used to assess functioning in neurodevelopmental disabilities (49). It provides an overall composite score of adaptive functioning, including subscores indexing social skills, conceptual and practical skills. Approximately 20 minutes to complete.

Scale to measure behavioural problems

1. Aberrant Behavioral Checklist-Irritability subscale (ABC-I): The ABC is a 58-item rating scale completed by a caregiver. It consists of 5 subscales. Among them, the Irritability subscale consists of 15 items. The ABC was empirically derived to assess treatment effects, and it has sound psychometric characteristics (50). Caregivers were asked to consider the participant's behaviour over the last four weeks at Screen/Baseline. Here we will only employ the ABC-I to confirm eligibility (presence of tantrums, aggression, self-injury) and to assess treatment effects (41). <10 minutes to complete.
2. Modified Overt Aggression Scale (MOAS): The MOAS (51) is a reliable measure used to measure behavioural problems in adults with IDD. It consisted of 4 items and is administered

by the researcher's interview with the caregiver. It was used as the primary outcome in one of the largest RCT on aggression in this population (52). <5 minutes to complete.

Scale to measure anxiety
General Anxiety Subscale of the Anxiety, Depression, and Mood Scale (ADAMS): The ADAMS (53) is an informant-report instrument specifically developed for adults with IDD, consisting of five subscales. Herein, we will only use the General anxiety subscale, which consists of seven items that cover five of the diagnostic criteria or symptoms of anxiety. The score on the General anxiety subscale ranges from 0 to 21. <5 minutes to complete.

Scales to measure overall clinical impression
Clinical Global Impressions (CGI) scale: The CGI is a well-established research rating tool to provide a brief, stand-alone assessment of the clinician's view of the patient's global functioning prior to and after initiating a study medication (54). The CGI-Severity (CGI-S) asks the clinician one question: "Considering your total clinical experience with this particular population, how mentally ill is the patient at this time?" which is rated on the following seven-point scale: 1=normal, not at all ill; 2=borderline mentally ill; 3=mildly ill; 4=moderately ill; 5=markedly ill; 6=severely ill; 7=among the most extremely ill patients. This rating is based upon the average severity of observed and reported symptoms, behaviour, and function in the past seven days. The CGI-Improvement (CGI-I) is for the clinician to compare the patient's overall clinical condition to the baseline visit and is rated on a seven-point scale: "Compared to the patient's condition at admission to the project [prior to medication initiation], this patient's condition is: 1=very much improved since the initiation of treatment; 2=much improved; 3=minimally improved; 4=no change from baseline (the initiation of treatment); 5=minimally worse; 6= much worse; 7=very much worse since the initiation of treatment." <5 minutes to complete.

Scales to assess capacity to make informed consent
MacArthur Competence Assessment Tool for Clinical Research (MacCAT-CR): The MacCAT-CR (44) provides a structured format for capacity assessment. Beginning with project-specific disclosures to potential participants, the MacCAT-CR measures the four generally accepted components of decision-making competence: understanding, appreciation, reasoning, and the ability to express a choice. The informed consent of the participants who fail to pass this assessment will be completed by their substitute decision maker. Herein, a score of 70% or higher in the MacCAT-CR will indicate that a given participant is deemed competent to consent. This cutoff is borrowed from the protocol of an ongoing Brain Canada-funded study on elderly with dementia - "Standardizing Care for Neuropsychiatric Symptoms and Quality of Life in Dementia". This cutoff of 70% is in-between the cut-offs used for schizophrenic adults (55) and neurotypical children (56), which we deem a middle ground to balance sensitivity and specificity of assessing capacity to consent. This should take approximately 15 minutes to complete.

In addition to using the MacCAT-CR scale, the research team will also utilize clinical assessment of the potential participants to determine their capacity to consent to participating in the research, in a similar manner to determining capacity for clinical treatments as outlined in clinical guidelines (45). During the research consent conversation, the research team member will look for evidence of the same four components of decision-making capacity. Specifically, research team members will use their clinical expertise and judgement to check that participants: (a) have an understanding

of what participating in this study entails and the associated benefits and risks; (b) have an appreciation for the how these benefits and risks apply to them specifically; (c) are able to show reasoning that their decision to participate aligns with their values; and (d) are able to communicate their choice to the research team. If during the consent conversation, the person obtaining consent finds, based on their clinical judgement, that any of these criteria are not found to be met, consent will have to be obtained by from the substitute decision maker.

Scales to assess adverse events related to the study medication

UKU side effect rating scale: The UKU side effect rating scale is a short and easy-to-use instrument that captures the core dimensions of side effects in patients using psychotropic medications. The UKU side effect rating scale is widely used in both research and in clinical settings (57), and is delivered by interviewing and observing the patients and their caregivers. Herein, we will adopt the UKU side effect rating scale specifically adjusted to adults with IDD, which consists of 35 items out of the original 48 items. This revised checklist seems more feasible to observe items that are concrete and objective than items based on the patients' subjective experiences (58). Approximately 20 minutes to complete.

Scales to measure caregiver's stress and crisis

1. The Stress subscale of Depression Anxiety Stress Scales (DASS-21): The 7-item Stress subscale of the original DASS-21 (59) is used to assess perceived feelings of parent stress. Items measure feelings experienced over the previous week and are scored on a 4-point Likert scale from 0 ("did not apply to me at all") to 3 ("applied to me very much, or most of the time"), providing total scores between 0 and 21 for stress. <5 minutes to complete

2. Brief Family Distress Scale (BFDS): The BFDS (60) measures the subjective experience of crisis in families of people with neurodevelopmental disabilities. This measure is meant to assess the experience from the caregiver's perspective and is placed along a continuum of distress, from mild stress to qualitatively distinct and immediate periods of crisis. <1 minute to complete.

Scales to measure acceptability

Custom-designed acceptability questionnaire: This questionnaire is similar to that used in (23), which inquires the acceptability of all study components, including recruitment, withdrawal rate, study visit attendance, protocol adherence and the time burden of completing questionnaires.

Other measures

1. Demographic questionnaire
2. Current and Concomitant medications (including type, dose, and duration of treatment)[*]
3. Height
4. Weight
5. Vital signs (blood pressure, heart rate, and temperature)
6. Drug diary to evaluate compliance

[*]The high dose of benzodiazepine (more than the benzodiazepine equivalent to lorazepam 2 mg daily) and other cannabinoids, which are still used 4 weeks before the start of the use of the study drug (titration phase), are not permitted. Other concurrent medications and non-medication treatments (including behavioural therapy, psychological therapy, occupational therapy, support group etc.) are permitted before and during the trial.

MR Imaging

MRI scans are optional and exploratory, dependent on the participants' capacity to comply with the procedures and the participants' and caregivers' willingness to participate. If the caregiver, substitute decision maker (SDM), and participants are willing and capable to receive the MRI assessment, they will receive an instruction using a custom-built social script, which comprises a series of photographs of the research procedures, environments, and files of the sounds emitted by MRI to help participants be acquainted with the scanning environment and learn the procedures at the screening visit (S-V). The visit of MRI assessments can be arranged either on the same day with the baseline assessment (V 0) or the other day before the start of the dose titration phase.

For the MRI measure, we will use the CAMH research-dedicated 3 Tesla MRI scanner, operated by a well-organized team including a full-time MR physicist and research technologist. The acquisitions we will obtain include a T1-weighted, a naturalistic film viewing fMRI, and proton magnetic resonance spectroscopy (^{1}H MRS) scans. The entire MR scan session will take approximately 30 minutes.

The acquisition details are as follows.

T1: A standard high-resolution T1-weighted scan will be acquired, as needed for processing both the fMRI and MRS data. Approximately 4.5 minutes to complete.

fMRI: High spatial and temporal resolution multiband EPI fMRI scans are acquired to examine functional activity. The task paradigm is using naturalistic film viewing, which will take place in the scanner. All participants are instructed to passively view the films and pay attention to the cinematic events. The negative-valence emotion clip is presented to the participants. The display of a film is preceded and followed by an epoch during which the participants passively gazed at an all-black slide. The duration of this epoch is 30 seconds (61). The naturalistic film stimuli are intentionally adopted as the paradigm for its ecologically valid nature. They approximate real life scenarios, which can elicit dynamic emotion responses and facilitate better participants' compliance in the scanner at the same time (62). The fear emotion clip is selected, as the negative emotion valence modulation is associated with the effect of THC on emotion regulation (20). Approximately 5 minutes to complete.

^{1}H MRS: A spectroscopy scan is based on the MEshcher-GArwood Point RESolved Spectroscopy (MEGA-PRESS) sequence. The neurochemical profile is extracted from the anterior cingulate cortex set as the volume of interest, according to our established parameters.
The cannabinoids help regulate excitatory glutamate (E) and inhibitory γ-aminobutyric acid (GABA) (I) transmission (13). Thus, we aim to use ^{1}H MRS to measure glutamate and GABA metabolite. The anterior cingulate cortex is chosen as the region of interest as it has a high CB1 concentration (20), and is critically involved in emotional regulation. Approximately 15 minutes to complete.

4. RECRUITMENT

The study team will inform the clinicians at the Adult Neurodevelopmental Services at CAMH and Surrey Place Centre about the study through an email introduction, followed by online meetings. Adult Neurodevelopmental Services, CAMH provide first-time intake psychiatric assessments to about 300 adults with IDD annually. 30% of them (around 90 people annually) may be potentially eligible patients. The treating physician/clinical care team will not obtain consent. They may identify potential research participants and obtain verbal permission from these potential participants and their SDM for a member of the research team to approach them. Potential participants and their substitute decision maker (SDM) who indicate further interest in hearing more about the study and provide assent to be contacted by a member of the research team will engage in an informed consent process (as described in the Telephone Screening Script).

CLEARR will be used to recruit participants for this study. CLEARR stands for Clinical Engagement And Research Recruitment. This has been approved by CAMH's Medical Advisory Committee, Information and Privacy Office and acknowledged by REB. All new referrals will be reviewed by the CLEARR coordinator and CLEARR physician for eligibility to participate using minimal inclusion/exclusion criteria outlined. Once a patient is identified, the attending physician will be notified via outlook calendar invite or email that their patient may be eligible for the study. The attending physician will decide whether research is appropriate for the patient and if so, they will ask the patient for consent to be contacted regarding the research study. If the patient provides verbal consent to be contacted to receive more information about the study, the physician will connect the patient with the CLEARR coordinator or study research team who will further explain the study. No PHI will be given to the study research team prior to the patient's consent.

Recruitment in the community (self-referral) will be conducted online via CAMH Research Registry, social media channels (Facebook, Twitter, Linkedin, Instagram) and community organizations providing services for individuals with IDD and their families. Advertisements may also be posted on relevant Facebook and Linkedin, or Instagram pages or be retweeted through their Twitter accounts. The message outlines the fundamentals of the study. Potential participants/SDM will then attend a research screening visit to determine eligibility if they meet all of the following criteria.

All potential participants and their SDM will be given the option to participate. Participation in the study is voluntary. The decision to participate will not affect patients' receipt of treatment or clinical services. Participants and their SDM will be informed that they have the option of terminating their participation at any time, without consequence and that no new data will be collected on them. Any existing data will be anonymized.

5. CONSENT

- The research assistant will schedule the first appointment (research screening visit) to discuss the study and obtain written informed consent from the participant or their SDM.
    - The participant or their SDM will be provided with the informed consent form either by email, mail, or secure file transfer (as noted in the Telephone Contact Script) before the consent discussion.

- SDM consent is included as the patient may not have the capacity to make the decision about study participation. As per the Tri-Council Policy Statement: Ethical Conduct for Research Involving Humans, principle of respect for persons implies that those who lack the capacity to make decisions for themselves should have the opportunity to participate in research that may be of benefit to themselves or others. SDMs may decide whether participation in a particular study would be appropriate. These principles involve considerations of concern for welfare and justice.
- Consent will be documented on the written Informed Consent Form (ICF). This will include signatures from the participant/SDM, the person conducting the consent discussion, and the witness (if applicable). Full explanation about this study will be provided to the participants and/or their SDMs.
    - If the consent discussion is conducted in person, the participant/SDM will be provided with a completed and signed copy of the informed consent form at the end of their visit.
- The MacArthur Competence Assessment Tool for Clinical Research (MacCAT-CR) will be applied to both of them as needed in order to ensure that they understand the information presented in the ICF. A score of 70% or higher in the MacCAT-CR will be required to pass.
    - 1) If the participant passes the MacCAT, written informed consent will be obtained from the participant.
    - 2) If the participant is not able to complete or does not pass the MacCAT and their SDM passes the MacCAT, written informed consent will be obtained from the SDM.
- If the participant and their SDM choose to have the screening assessment and consent discussion virtually implemented (an elective process), they will receive the informed consent form either by email, mail, or secure file transfer (as noted in the Telephone Contact Script) before the consent discussion. If the participant/SDM chooses to receive the informed consent form by mail, after virtually conducting the consent discussion, we will document remote consent by acquiring a written signature from the prospective participant or their SDM on the paper informed consent form and have it mailed back to the research team using a pre-paid postage envelope. The research team member who conducted the consent discussion would then sign this informed consent form. Alternately, if the participant/SDM chooses to receive the informed consent form by email or secure file transfer, the participant/SDM will print and sign the informed consent form, and mail the completed form with their wet signatures back to the study team. If participants/SDMs choose to return the signed informed consent form by mail, we will provide a pre-paid postage envelope.
    - During videoconferencing/teleconferencing, we will assess capacity and address any questions raised by prospective participants/SDMs prior to documenting informed consent, as identical to a consent discussion conducted in person.

- The participant/SDM will receive a fully signed, complete copy of the informed consent form within 10 days following the informed consent process. This complete copy will be distributed by mail.
- The research procedures will only begin after the informed consent form signed by the participant/SDM (wet signature) is received and signed by the research team member who conducted the consent discussion.

6. STUDY DURATION

The estimated length of time needed to complete the entire study is 2 years, including 3 months of start-up preparation, 18 months of participants recruitment and trials, and 3 months of data analysis and final reporting.

7. STOPPING OF THE STUDY

7.1. Termination of the entire study

Reasons for withdrawing individual participants from the study may include one or more of the following:

a) Failure to continue to meet the inclusion criteria.
b) Changes in participants' behaviours or situations, which meet the exclusion criteria. For example, participants become pregnant, newly use other cannabinoids, or newly use illegal psychomimetic substances as specified in Section 3.3, after the start of the trial.
c) Major protocol violation.
d) Participant lost to follow-up.
e) Withdrawal of consent: Participation in the study is voluntary, and the participant can refuse or stop participation at any time. The participant's refusal to participate or withdraw from the study will not interfere in any way with the relationship with his/her doctor or hospital.
f) Inability to tolerate the study medication or procedure.
g) Serious adverse effects related to the study medication.
h) New information shows that the study intervention is no longer in the participant's best interest.

In addition to these discontinuation criteria, any participant may be discontinued from the study at the discretion of the investigators if this is deemed to be in the best interest of the participant. The decision may be made either to protect the participant's health and safety, or because it is part of the research plan that people who develop certain conditions may not continue to participate. To ensure safety, we will arrange a Safety Follow-up visit, which is the same as the one for those who fully complete the trials, for participants withdrawn from the study.

Any research information recorded for, or resulting from, participation in this research study prior to the date that the participant formally withdraws from the study will be retained and may

continue to be used and disclosed by the investigators for research purposes. However, no new data will be further collected. There is no exception to the Safety Follow-up visit.

As the purpose of this phase I study is to investigate the tolerability of nabilone in adults with IDD, we will not recruit any new participants to replace the ones who withdraw from the entire trial.

7.2. Stopping of parts of the study

If participants fail to discontinue the use of any cannabinoids or high doses of benzodiazepine (more than the benzodiazepine equivalent to lorazepam 2 mg daily), psychostimulants for attention-deficit hyperactivity disorder (ADHD), and nonbenzodiazepine hypnotics 4 weeks before the start of the titration phase, they will not progress to the Pre-Treatment visit (V 0). We will discuss with participants and their caregivers to confirm their willingness to further proceed with the study. The participants are only permitted to proceed with the trial when they do not fulfill this exclusion criterion of the concurrent use of other cannabinoids and high dose of benzodiazepine.

If participants cannot, or are unwilling to, comply with the procedures of NIH Toolbox® Cognition Battery, but can comply with the remaining parts of the study, we will stop assessing them using this cognitive battery. These participants will still proceed with the other parts of the entire studies.

8. SAMPLE SIZE AND DATA ANALYSIS

To test the Hypotheses 1 and 2, we aim to enroll 40 patients to enter the dose titration phase, consistent with common sample sizes for Phase I trials (63).
We assume the attrition rate of 25% from the dose titration phase to entering the open-label phase. To test behavioural changes at the group level (Exploratory Objective), the sample size of the remaining participants (N=30) could be sufficient to detect differences with effect size Cohen's d as low as of 0.53, and at power = 0.8 and two-sided α-level = 0.05 (G*Power 3.1), which is similar to that detected in the study investigating the effects of nabilone on agitation associated with dementia (27).
Paired-t test will be used to investigate the pre- vs. post-treatment behavioural and brain changes. Exploratory analyses will be performed to determine predictors of response including baseline individual features, using logistic regression. Safety profiles will be described in the percentage of each adverse effects. For MRI measures, non-parametric tests will be used to explore the brain changes with nabilone treatment, assuming that only few participants can finish MRI scans eventually.

9. POTENTIAL RISKS

9.1. From questionnaires and cognitive testing

Participants may become tired, and if so will be able to take rest breaks or split the visits. Some of the assessments, in particular the questionnaires, may be upsetting to study participants. We will minimize all risks by having assessments performed by well-trained research staff with clinical

experience and appropriate skills to maximize the participants' comfort and keep their distress to a minimum during each visit.

9.2. From MRI (optional)

General Risks: MRI is a non-invasive procedure, although there are contraindications such as having a pacemaker, and having ferrous implants. For this reason, all participants will be screened using the current MRI Safety Screening form. The major discomfort with MRI scanning is a knocking sound that the machine makes; earplugs will be available to decrease the noise. A microphone will be provided so that they may be able to communicate and stop the testing if they become too uncomfortable or anxious at any time.

Psychological distress from MRI: Some subjects can experience claustrophobia while in the scanner. To minimize the potential distress from unfamiliarity, the participants will receive an instruction using a custom-built social script, which comprises a series of photographs of the research procedures, environments, and files of the sounds emitted by MRI to help participants be acquainted with the scanning environment and learn the procedures at the screening visit (S-V). Participants will have the opportunity to examine this space or the mock scanner before the scanning starts. Mild psychological distress must also be considered in these studies. It is not advisable for subjects to involve themselves in this study if they show a fearful behaviour to enclosed spaces or if they have a history of not tolerating a MRI scan.
It is also possible that the participants may find the scanning procedures stressful. They will be able to discuss any concerns with study staff at any length at any time. On scan days, study staff will be available at all times, including during the scan (via call button and microphone in the scanner room). In addition, medical staff will be on call if participants/SDMs want to discuss matters with a doctor.

Risk of Incidental Findings: The possibility of unexpected or incidental findings carries with it some risks. Research scans are not designed to be used for diagnosis. In the unlikely event an atypical finding is seen on this optional MRI scan, we may ask a radiologist or other qualified health professional to look at this participant scan, which will be specified in the MRI consent form. The participant's identity will not be revealed to the radiologist. If the qualified professional recommends further tests to determine the nature and significance of any incidental findings on the specific MRI scan, we will contact the participant or his/her SDM to help arrange medical follow-up.

9.3. From nabilone

Nabilone is indicated for severe nausea and vomiting associated with cancer chemotherapy and commercially available in Canada. Its use is usually safe and well tolerated, with sedation (~20%) and dry mouth (~10%) being the most commonly reported adverse events (no side effects of appetite and movement) in elderly with dementia or Parkinson's disease, or people with cancer (26-28, 30, 34). This safety profile is comparable to those reported in studies of CBD (36) and clonidine (37) (one of the medications for SBP of people with developmental disabilities) use in people with IDD, and less substantial relative to antipsychotics.
The addictive potential of nabilone is very low and unlikely (38), for its effect of less euphoria, slower onset of action, and more difficult titration compared to smoking cannabis. Nabilone has been shown to help with sustained abstinence from marijuana (39). The incidence of nabilone

induced full-blown psychotic disorder are also rare, and this propensity is only concerning in people with a personal or family history of schizophrenia, who will be excluded from this study. Although promising, no data ever showing efficacy and safety regarding nabilone in this population have been noted.

Per the product monograph and previous reports issued by the Canadian Agency for Drugs and Technologies in Health (26-28, 30, 34, 35), risks and side effects related to nabilone we are studying include:

- Drowsiness (66.0%)
- Vertigo (58.8%)
- Psychological high (38.8%)
- Dry mouth (21.6%)
- Depression (14.0%)
- Ataxia (reduced body coordination) (12.8%)
- Blurred vision (12.8%)
- Sensation disturbance (12.4%)
- Anorexia (loss of appetite) (7.6%)
- Asthenia (feeling of numbness and tingling) (7.6%)
- Headache (7.2%)
- Orthostatic hypotension (feeling dizzy when standing up) (5.2%)
- Euphoria (4.0%)
- Hallucinations (2.0%).

However, these estimates are influenced by factors such as drug dose, patient characteristics, detection technique, setting, and physician judgments, among others. Consequently, the side effects listed above are presented solely to indicate the relative frequency of adverse events reported in representative controlled clinical studies conducted to evaluate the safety and efficacy of nabilone across different situations.

10. POTENTIAL BENEFITS OF THE PROPOSED RESEARCH TO THE PARTICIPANTS AND OTHERS

The risks in the study appear reasonable in terms of the potential benefits of knowledge leading to the amelioration/prevention of the extraordinarily debilitating effects of a major mental illness. Participants will be informed that no individual benefits will likely accrue other than the participant's honorarium. Of course, should an unsuspected abnormality be detected, participants will be notified, but the chances of this benefit occurring as a result of this study are small.

The investigators responsible for this study or the CAMH are not conducting this study to receive commercial benefit. However, if this research produces financial returns from the commercialization of the results in the future, participants will not receive any benefit from these returns.

If participants request feedback about results, one of the co-investigators or the research coordinator will meet with the participant to give feedback.

11. DATA MANAGEMENT, RETENTION AND INTEGRITY

The basic protection against risk in this study will be provided by Dr. Lin (study PI and QI). He will have primary responsibility for the monitoring of participants during the entire time they participate in the study. PI will meet weekly with study personnel to review accrued data, data confidentiality, and adherence to protocol design, recruitment, and participant complaints. During meetings, the study PI will also review the enrolment data, the accrual and integrity of neuroimaging, clinical, and neurocognitive data, and any adverse event associated with the various components of the study. Based on these reports, we will determine if there has been any change in the benefit-to-risk ratio of the neuroimaging and clinical and cognitive assessment components of the study.

All data pertaining to a participant's involvement in this study will be coded and stored in locked offices. This information will only be accessible to the research team. In unusual cases, a participant's research records may be released in response to a court order. If the research team learns that a participant or someone with whom the participant is involved with is in serious danger or harm, an investigator will inform the appropriate agencies as per legal or regulatory requirements.

MRI scans (optional) will be anonymized by using only the participant study IDs and the date and time of scanning and will be stored on a secure server. All imaging data, derived measures, and demographic data will be accessible via webportal (hosted at CAMH) through unique username and password credentials. Results will be published as group data without the use of characteristics that would identify individual participants.

The hard data are stored in a locked filing cabinet stored in a locked office to further protect participant anonymity. Data auditing, entry and quality control will be carried out regularly. Regularly scheduled, and as needed, communications between the study team, the neuroimaging team and PI will clarify any inconsistencies and ambiguities in the data.

Study data will be entered in a secure database (REDCap). At point-of-entry, data values will undergo consistency edits (e.g., ID validation, range verification, duplicate detection) and personnel will be required to correct errors. Data management staff will run logic error programs to check for accuracy and irregularities within and across data structures and within and across sites. Quality assurance checks will be conducted daily by site personnel, as well as biweekly by data management staff.

During the duration of the trial, data and all appropriate documentation will be maintained according to current regulations, and stored for a minimum of 25 years after the completion of the study, including the follow-up period.

12. SAFETY MONITORING PLAN FOR ADVERSE EVENTS AND SEVERE ADVERSE EVENTS

Adverse events (AEs) will be documented and reported as mandated by current regulations. AEs will be assessed at each study visit and telephone follow-up. All AEs, whether reported by the participant, SDM, or observed by study staff/investigators, will be recorded on the AE log along with a brief description, start date/resolution date and any action taken. The AE log will be initiated by the PI (Dr. Lin), who will make the determination on the relationship of the AE to the investigational drug/study procedures. All unexpected AEs determined by the PI to have a causal relationship with the investigational product or as a result of study procedures, will be reported to CAMH REB as part of the annual ethics approval renewal process. Where the event is deemed to meet CAMH REB reporting criteria, the PI will notify the REB. Every adverse event will be assessed and recorded in the participant's file by the PI or another physician involved in the study. The PI will report all Serious and Unexpected Adverse Drug Reactions (SUADR) to Health Canada as per Division 5 regulations.

If participants have AEs from participation in this study, medical care will be provided in the same way they would normally get medical care (for example, by going to their family doctor or seeking emergency medical treatment if needed). If participants withdraw from the study because they are unable to tolerate any AEs, or because of serious AEs, we will arrange a Safety Follow-up visit, which is the same as the one for those who fully complete the trials. If participants can tolerate the AEs, and the investigators consider that the continuation of participation in the study is still in the best interest of these participants, the frequency of the regular phone check-up during the open label phase will be increased from once weekly to once every two days to make sure these AEs do not progress to intolerable or more serious ones. In addition to intolerable AEs/severe AEs, there are other criteria which will remove participants from continuing the study (see Section 7.1).

Nonetheless, we do not have an independent Data and Safety Monitoring Board (DSMB) in the current trial for the following reasons:

This proposal is a study that involves single data collection site (CAMH), unblinded data, short-term data collection, small numbers of participants (N = 30), and clear criteria regarding removing participants from the trial. This study of short-term intervention with small numbers of participants cannot generate enough data for interim analyses to permit the establishment of an effective study-wise stopping rule. Moreover, this proposal is the investigator-initiated trial and there are no biomedical or financial conflicts of interest regarding using Nabilone or any cannabinoids to treat individuals with IDD within the research team. Lastly, based on the published data, the possible adverse outcomes for participants in the current trial appears moderate (temporary, non-life-threatening conditions).

These rationales conform to Tri-Council Policy Statement: Ethical Conduct for Research Involving Humans – TCPS 2 (2018) – Chapter 11: Clinical Trials 11.7 (https://ethics.gc.ca/eng/tcps2-eptc2_2018_chapter11-chapitre11.html#7).

13. STUDY MANAGEMENT AND MATERIALS

CAMH Investigators will retain a participant identification code list if they need to contact participants after the study. This list will contain the complete name, identification number, address and phone number of all participants and will be held confidentially at the investigators site after completion of the study.

Study data will be entered into a secure database (REDCap). An eCRF will be completed for each participant enrolled in the study. A participant screening log, noting reasons for screen failure, where applicable, will be maintained for all participants. The investigator will document the obtained informed consent and record all medication administration (including concomitant medication(s)), medical history, and efficacy data in the eCRF. Psychopathology scales and neuropsychological assessments will be considered source documents and will be incorporated into the eCRF in a confidential manner.

14. CONFIDENTIALITY

There is a potential risk of breach of confidentiality that is inherent in all research protocols. Breach of confidentiality will be minimized by the staff who will maintain research data (identified only by participant code number not related to name, or date of birth) in separate charts and a dedicated password protected electronic database. A list of participant names, their ID numbers, and information about how they can be reached will be kept in a separate locked cabinet with access only to study personnel authorized by the PI. Procedures have been established, and will be followed, to minimize the risk of breach of confidentiality. Procedures to maintain confidentially include: (1) formal training sessions for all research staff emphasizing the importance of confidentiality; (2) specific procedures developed to protect participants' confidentiality, and (3) formal mechanisms limiting access to information that can link data to individual participants. All information obtained from participants will be kept as confidential as possible. Computer based files/data will be entered into password-secured databases and paper-based files will be stored in a secure location. These data will only be accessible to personnel involved in the study, and they will abide by confidentiality regulations of the REB. The ethics committee will be granted direct access to the study participants' original medical records for verification of study procedures and/or data, without violating the confidentiality of the participants, to the extent permitted by the law .

The brain imaging information collected as part of this study may be shared on public scientific repositories (e.g. openfmri.org). In this case, study IDs associated with the MRI scans will be changed to a newly anonymized ID. Any identifying information in the scan data will be strictly stripped; for example any portion of the scans that might allow an image of the participant's face will be removed (so called "defacing"). In addition to the scans, basic demographic information (e.g. age and gender) and some summary clinical scores collected may be shared. This information will be shared in order to make the best possible scientific use of the information collected as part of this study, for example by allowing other researchers around the world conducting similar research to directly compare their results to what we collect at CAMH. This also allows for greater transparency in research, where other researchers can directly examine information collected across studies to ensure the results are correct. Data will not be added to

repositories until the study is completed. This data will be anonymized and not contain any personal health information.

Participants will not be identified by name in any publication of research results. Results will be published as group data without the use of characteristics that would identify individual participants.

15. FINANCIAL COMPENSATION

Study participants will receive a stipend of $200.00 (280 if MRI) to compensate them for their time after they complete all the procedures of the study ($20/hour for the on-site visit; 2 additional hours for each optional MRI scan). However, if they drop out of the study or their participation is terminated, they will be reimbursed at the time of the last scheduled visit based on what they have completed.

16. REFERENCES

1. Morris SP, Fawcett G, Brisebois L, Hughes J: A demographic, employment and income profile of Canadians with disabilities aged 15 years and over, 2017, Statistics Canada= Statistque Canada; 2018.
2. Cooper SA, van der Speck R. Epidemiology of mental ill health in adults with intellectual disabilities. Curr Opin Psychiatry. 2009;22:431-436.
3. de Winter CF, Jansen AA, Evenhuis HM. Physical conditions and challenging behaviour in people with intellectual disability: a systematic review. J Intellect Disabil Res. 2011;55:675-698.
4. Dawson F, Shanahan S, Fitzsimons E, O'Malley G, Mac Giollabhui N, Bramham J. The impact of caring for an adult with intellectual disability and psychiatric comorbidity on carer stress and psychological distress. J Intellect Disabil Res. 2016;60:553-563.
5. National Collaborating Centre for Mental H: National Institute for Health and Care Excellence: Clinical Guidelines. in Challenging Behaviour and Learning Disabilities: Prevention and Interventions for People with Learning Disabilities Whose Behaviour Challenges. London, National Institute for Health and Care Excellence (UK)
Copyright © The British Psychological Society & The Royal College of Psychiatrists, 2015.; 2015.
6. Sullivan WF, Diepstra H, Heng J, Ally S, Bradley E, Casson I, Hennen B, Kelly M, Korossy M, McNeil K, Abells D, Amaria K, Boyd K, Gemmill M, Grier E, Kennie-Kaulbach N, Ketchell M, Ladouceur J, Lepp A, Lunsky Y, McMillan S, Niel U, Sacks S, Shea S, Stringer K, Sue K, Witherbee S. Primary care of adults with intellectual and developmental disabilities: 2018 Canadian consensus guidelines. Canadian family physician Medecin de famille canadien. 2018;64:254-279.
7. Lunsky Y, Khuu W, Tadrous M, Vigod S, Cobigo V, Gomes T. Antipsychotic Use With and Without Comorbid Psychiatric Diagnosis Among Adults with Intellectual and Developmental Disabilities. Canadian journal of psychiatry Revue canadienne de psychiatrie. 2018;63:361-369.
8. Lunsky Y, Modi M. Predictors of Psychotropic Polypharmacy Among Outpatients With Psychiatric Disorders and Intellectual Disability. Psychiatric services (Washington, DC). 2018;69:242-246.

9. Deutsch SI, Burket JA. Psychotropic medication use for adults and older adults with intellectual disability; selective review, recommendations and future directions. Progress in neuro-psychopharmacology & biological psychiatry. 2021;104:110017.
10. Vereenooghe L, Flynn S, Hastings RP, Adams D, Chauhan U, Cooper SA, Gore N, Hatton C, Hood K, Jahoda A, Langdon PE, McNamara R, Oliver C, Roy A, Totsika V, Waite J. Interventions for mental health problems in children and adults with severe intellectual disabilities: a systematic review. BMJ Open. 2018;8:e021911.
11. Sheehan R, Horsfall L, Strydom A, Osborn D, Walters K, Hassiotis A. Movement side effects of antipsychotic drugs in adults with and without intellectual disability: UK population-based cohort study. BMJ Open. 2017;7:e017406.
12. Vancampfort D, Schuch F, Van Damme T, Firth J, Suetani S, Stubbs B, Van Biesen D. Metabolic syndrome and its components in people with intellectual disability: a meta-analysis. J Intellect Disabil Res. 2020;64:804-815.
13. Murillo-Rodriguez E, Pastrana-Trejo JC, Salas-Crisóstomo M, de-la-Cruz M. The Endocannabinoid System Modulating Levels of Consciousness, Emotions and Likely Dream Contents. CNS & neurological disorders drug targets. 2017;16:370-379.
14. Capuron L, Castanon N. Role of Inflammation in the Development of Neuropsychiatric Symptom Domains: Evidence and Mechanisms. Current topics in behavioral neurosciences. 2017;31:31-44.
15. Krefft M, Frydecka D, Zalsman G, Krzystek-Korpacka M, Śmigiel R, Gębura K, Bogunia-Kubik K, Misiak B. A pro-inflammatory phenotype is associated with behavioural traits in children with Prader-Willi syndrome. European child & adolescent psychiatry. 2020.
16. Iuvone T, Esposito G, Esposito R, Santamaria R, Di Rosa M, Izzo AA. Neuroprotective effect of cannabidiol, a non-psychoactive component from Cannabis sativa, on beta-amyloid-induced toxicity in PC12 cells. Journal of neurochemistry. 2004;89:134-141.
17. Kolla NJ, Mishra A. The Endocannabinoid System, Aggression, and the Violence of Synthetic Cannabinoid Use, Borderline Personality Disorder, Antisocial Personality Disorder, and Other Psychiatric Disorders. Frontiers in behavioral neuroscience. 2018;12:41.
18. Hosie S, Malone DT, Liu S, Glass M, Adlard PA, Hannan AJ, Hill-Yardin EL. Altered Amygdala Excitation and CB1 Receptor Modulation of Aggressive Behavior in the Neuroligin-3(R451C) Mouse Model of Autism. Frontiers in cellular neuroscience. 2018;12:234.
19. Kirtley OJ, O'Carroll RE, O'Connor RC. The role of endogenous opioids in non-suicidal self-injurious behavior: methodological challenges. Neuroscience and biobehavioral reviews. 2015;48:186-189.
20. Bloomfield MAP, Hindocha C, Green SF, Wall MB, Lees R, Petrilli K, Costello H, Ogunbiyi MO, Bossong MG, Freeman TP. The neuropsychopharmacology of cannabis: A review of human imaging studies. Pharmacol Ther. 2019;195:132-161.
21. Hasler G, Buchmann A, Haynes M, Müller ST, Ghisleni C, Brechbühl S, Tuura R. Association between prefrontal glutamine levels and neuroticism determined using proton magnetic resonance spectroscopy. Translational psychiatry. 2019;9:170.
22. Hess P: Cannabis and autism, explained. 2020.
23. Efron D, Taylor K, Payne JM, Freeman JL, Cranswick N, Mulraney M, Prakash C, Lee KJ, Williams K. Does cannabidiol reduce severe behavioural problems in children with intellectual disability? Study protocol for a pilot single-site phase I/II randomised placebo controlled trial. BMJ Open. 2020;10:e034362.

24. Abramovici H: Information for Health Care Professionals: Cannabis (marihuana, marijuana) and the cannabinoids. Ottawa: Health Canada, 2013. 2018.
25. Elsaid S, Kloiber S, Le Foll B. Effects of cannabidiol (CBD) in neuropsychiatric disorders: A review of pre-clinical and clinical findings. Progress in molecular biology and translational science. 2019;167:25-75.
26. CADTH Rapid Response Reports. in Long-term Nabilone Use: A Review of the Clinical Effectiveness and Safety. Ottawa (ON), Canadian Agency for Drugs and Technologies in Health Copyright © 2015 Canadian Agency for Drugs and Technologies in Health.; 2015.
27. Herrmann N, Ruthirakuhan M, Gallagher D, Verhoeff N, Kiss A, Black SE, Lanctôt KL. Randomized Placebo-Controlled Trial of Nabilone for Agitation in Alzheimer's Disease. The American journal of geriatric psychiatry : official journal of the American Association for Geriatric Psychiatry. 2019;27:1161-1173.
28. Hillen JB, Soulsby N, Alderman C, Caughey GE. Safety and effectiveness of cannabinoids for the treatment of neuropsychiatric symptoms in dementia: a systematic review. Therapeutic advances in drug safety. 2019;10:2042098619846993.
29. Black N, Stockings E, Campbell G, Tran LT, Zagic D, Hall WD, Farrell M, Degenhardt L. Cannabinoids for the treatment of mental disorders and symptoms of mental disorders: a systematic review and meta-analysis. The lancet Psychiatry. 2019;6:995-1010.
30. Peball M, Krismer F, Knaus HG, Djamshidian A, Werkmann M, Carbone F, Ellmerer P, Heim B, Marini K, Valent D, Goebel G, Ulmer H, Stockner H, Wenning GK, Stolz R, Krejcy K, Poewe W, Seppi K. Non-Motor Symptoms in Parkinson's Disease are Reduced by Nabilone. Annals of neurology. 2020.
31. Kruger T, Christophersen EJJoD, Pediatrics B. An open label study of the use of dronabinol (Marinol) in the management of treatment-resistant self-injurious behavior in 10 retarded adolescent patients. Cannabinoids. 2006;27:433.
32. Kurz R, Blaas KJC. Use of dronabinol (delta-9-THC) in autism: a prospective single-case-study with an early infantile autistic child. J Dev Behav Pediatr. 2010;5:4-6.
33. Bedi G, Cooper ZD, Haney M. Subjective, cognitive and cardiovascular dose-effect profile of nabilone and dronabinol in marijuana smokers. Addict Biol. 2013;18:872-881.
34. Cowling T, MacDougall D: CADTH Rapid Response Reports. in Nabilone for the Treatment of Post-Traumatic Stress Disorder: A Review of Clinical Effectiveness and Guidelines. Ottawa (ON), Canadian Agency for Drugs and Technologies in Health Copyright © 2019 Canadian Agency for Drugs and Technologies in Health.; 2019.
35. Teva Canada Limited: TEVA-NABILONE (Product Monograph). Edited by Teva Canada Limited. Canada2012.
36. Devinsky O, Cross JH, Laux L, Marsh E, Miller I, Nabbout R, Scheffer IE, Thiele EA, Wright S, Cannabidiol in Dravet Syndrome Study G. Trial of Cannabidiol for Drug-Resistant Seizures in the Dravet Syndrome. N Engl J Med. 2017;376:2011-2020.
37. Agarwal V, Sitholey P, Kumar S, Prasad M. Double-blind, placebo-controlled trial of clonidine in hyperactive children with mental retardation. Mental retardation. 2001;39:259-267.
38. Ware MA, St Arnaud-Trempe E. The abuse potential of the synthetic cannabinoid nabilone. Addiction (Abingdon, England). 2010;105:494-503.
39. Haney M, Cooper ZD, Bedi G, Vosburg SK, Comer SD, Foltin RW. Nabilone decreases marijuana withdrawal and a laboratory measure of marijuana relapse. Neuropsychopharmacology : official publication of the American College of Neuropsychopharmacology. 2013;38:1557-1565.

40. Health Canada: Clinical trials and drug safety. 2020.
41. Aman MG, McDougle CJ, Scahill L, Handen B, Arnold LE, Johnson C, Stigler KA, Bearss K, Butter E, Swiezy NB, Sukhodolsky DD, Ramadan Y, Pozdol SL, Nikolov R, Lecavalier L, Kohn AE, Koenig K, Hollway JA, Korzekwa P, Gavaletz A, Mulick JA, Hall KL, Dziura J, Ritz L, Trollinger S, Yu S, Vitiello B, Wagner A, Research Units on Pediatric Psychopharmacology Autism N. Medication and parent training in children with pervasive developmental disorders and serious behavior problems: results from a randomized clinical trial. J Am Acad Child Adolesc Psychiatry. 2009;48:1143-1154.
42. Shields RH, Kaat AJ, McKenzie FJ, Drayton A, Sansone SM, Coleman J, Michalak C, Riley K, Berry-Kravis E, Gershon RC, Widaman KF, Hessl D. Validation of the NIH Toolbox Cognitive Battery in intellectual disability. Neurology. 2020;94:e1229-e1240.
43. Oliver C, Richards C. Practitioner Review: Self-injurious behaviour in children with developmental delay. J Child Psychol Psychiatry. 2015;56:1042-1054.
44. Appelbaum PS, Grisso T: MacArthur competence assessment tool for clinical research (MacCAT-CR), professional resource press/professional resource exchange; 2001.
45. Barstow C, Shahan B, Roberts M. Evaluating Medical Decision-Making Capacity in Practice. American family physician. 2018;98:40-46.
46. Moss S, Ibbotson B, Prosser H, Goldberg D, Patel P, Simpson N. Validity of the PAS-ADD for detecting psychiatric symptoms in adults with learning disability (mental retardation). Social psychiatry and psychiatric epidemiology. 1997;32:344-354.
47. Derks O, Heinrich M, Brooks W, Sterkenburg P, McCarthy J, Underwood L, Sappok T. The Social Communication Questionnaire for adults with intellectual disability: SCQ-AID. Autism Res. 2017;10:1481-1490.
48. Weintraub S, Dikmen SS, Heaton RK, Tulsky DS, Zelazo PD, Slotkin J, Carlozzi NE, Bauer PJ, Wallner-Allen K, Fox N, Havlik R, Beaumont JL, Mungas D, Manly JJ, Moy C, Conway K, Edwards E, Nowinski CJ, Gershon R. The cognition battery of the NIH toolbox for assessment of neurological and behavioral function: validation in an adult sample. J Int Neuropsychol Soc. 2014;20:567-578.
49. Kenworthy L, Case L, Harms MB, Martin A, Wallace GL. Adaptive behavior ratings correlate with symptomatology and IQ among individuals with high-functioning autism spectrum disorders. J Autism Dev Disord. 2010;40:416-423.
50. Aman MG, Singh NN, Stewart AW, Field CJ. Psychometric characteristics of the aberrant behavior checklist. Am J Ment Defic. 1985;89:492-502.
51. Oliver P, Crawford M, Rao B, Reece B, Tyrer PJJoARiID. Modified Overt Aggression Scale (MOAS) for people with intellectual disability and aggressive challenging behaviour: a reliability study. 2007;20:368-372.
52. Tyrer P, Oliver-Africano PC, Ahmed Z, Bouras N, Cooray S, Deb S, Murphy D, Hare M, Meade M, Reece B, Kramo K, Bhaumik S, Harley D, Regan A, Thomas D, Rao B, North B, Eliahoo J, Karatela S, Soni A, Crawford M. Risperidone, haloperidol, and placebo in the treatment of aggressive challenging behaviour in patients with intellectual disability: a randomised controlled trial. Lancet. 2008;371:57-63.
53. Esbensen AJ, Rojahn J, Aman MG, Ruedrich S. Reliability and validity of an assessment instrument for anxiety, depression, and mood among individuals with mental retardation. J Autism Dev Disord. 2003;33:617-629.
54. Busner J, Targum SD. The clinical global impressions scale: applying a research tool in clinical practice. Psychiatry (Edgmont (Pa : Township)). 2007;4:28-37.

55. Moser DJ, Schultz SK, Arndt S, Benjamin ML, Fleming FW, Brems CS, Paulsen JS, Appelbaum PS, Andreasen NC. Capacity to provide informed consent for participation in schizophrenia and HIV research. The American journal of psychiatry. 2002;159:1201-1207.
56. Hein IM, Troost PW, Lindeboom R, Benninga MA, Zwaan CM, van Goudoever JB, Lindauer RJ. Accuracy of the MacArthur competence assessment tool for clinical research (MacCAT-CR) for measuring children's competence to consent to clinical research. JAMA pediatrics. 2014;168:1147-1153.
57. Lingjaerde O, Ahlfors UG, Bech P, Dencker SJ, Elgen K. The UKU side effect rating scale. A new comprehensive rating scale for psychotropic drugs and a cross-sectional study of side effects in neuroleptic-treated patients. Acta psychiatrica Scandinavica Supplementum. 1987;334:1-100.
58. Tveter AL, Bakken TL, Bramness JG, Røssberg JIJAiMH, Disabilities I. Adjustment of the UKU Side Effect Rating Scale for adults with intellectual disabilities. A pilot study. 2014.
59. Henry JD, Crawford JR. The short-form version of the Depression Anxiety Stress Scales (DASS-21): construct validity and normative data in a large non-clinical sample. The British journal of clinical psychology. 2005;44:227-239.
60. Weiss JA, Lunsky YJJoC, Studies F. The brief family distress scale: A measure of crisis in caregivers of individuals with autism spectrum disorders. 2011;20:521-528.
61. Raz G, Touroutoglou A, Wilson-Mendenhall C, Gilam G, Lin T, Gonen T, Jacob Y, Atzil S, Admon R, Bleich-Cohen M, Maron-Katz A, Hendler T, Barrett LF. Functional connectivity dynamics during film viewing reveal common networks for different emotional experiences. Cogn Affect Behav Neurosci. 2016;16:709-723.
62. Sonkusare S, Breakspear M, Guo C. Naturalistic Stimuli in Neuroscience: Critically Acclaimed. Trends Cogn Sci. 2019;23:699-714.
63. Dahlberg SE, Shapiro GI, Clark JW, Johnson BE. Evaluation of statistical designs in phase I expansion cohorts: the Dana-Farber/Harvard Cancer Center experience. J Natl Cancer Inst. 2014;106.
